# New insights into the targeting of a subset of tail-anchored proteins to the outer mitochondrial membrane

**DOI:** 10.3389/fpls.2014.00426

**Published:** 2014-09-04

**Authors:** Naomi J. Marty, Howard J. Teresinski, Yeen Ting Hwang, Eric A. Clendening, Satinder K. Gidda, Elwira Sliwinska, Daiyuan Zhang, Ján A. Miernyk, Glauber C. Brito, David W. Andrews, John M. Dyer, Robert T. Mullen

**Affiliations:** ^1^Department of Molecular and Cellular Biology, University of GuelphGuelph, ON, Canada; ^2^Department of Plant Genetics, Physiology and Biotechnology, University of Technology and Life Sciences in BydgoszczBydgoszcz, Poland; ^3^United States Department of Agriculture, Agricultural Research Service, US Arid-Land Agricultural Research CenterMaricopa, AZ, USA; ^4^United States Department of Agriculture, Agricultural Research Service, Plant Genetics Research Unit, University of MissouriColumbia, MO, USA; ^5^Instituto do Cancer do Estado de Sao Paulo, Fundacao Faculdade de Medicina, Universidade de Sao PauloSao Paulo, Brazil; ^6^Sunnybrook Research Institute and Department of Biochemistry, University of TorontoToronto, ON, Canada

**Keywords:** *A. thaliana*, dibasic motif, mitochondria, outer mitochondrial membrane, tail anchored, targeting signal

## Abstract

Tail-anchored (TA) proteins are a unique class of functionally diverse membrane proteins defined by their single C-terminal membrane-spanning domain and their ability to insert post-translationally into specific organelles with an N_cytoplasm_-C_organelle interior_ orientation. The molecular mechanisms by which TA proteins are sorted to the proper organelles are not well-understood. Herein we present results indicating that a dibasic targeting motif (i.e., -R-R/K/H-X^{X≠E}^) identified previously in the C terminus of the mitochondrial isoform of the TA protein cytochrome *b*_5_, also exists in many other *A. thaliana* outer mitochondrial membrane (OMM)-TA proteins. This motif is conspicuously absent, however, in all but one of the TA protein subunits of the translocon at the outer membrane of mitochondria (TOM), suggesting that these two groups of proteins utilize distinct biogenetic pathways. Consistent with this premise, we show that the TA sequences of the dibasic-containing proteins are both necessary and sufficient for targeting to mitochondria, and are interchangeable, while the TA regions of TOM proteins lacking a dibasic motif are necessary, but not sufficient for localization, and cannot be functionally exchanged. We also present results from a comprehensive mutational analysis of the dibasic motif and surrounding sequences that not only greatly expands the functional definition and context-dependent properties of this targeting signal, but also led to the identification of other novel putative OMM-TA proteins. Collectively, these results provide important insight to the complexity of the targeting pathways involved in the biogenesis of OMM-TA proteins and help define a consensus targeting motif that is utilized by at least a subset of these proteins.

## Introduction

Tail-anchored (TA) proteins are a unique class of proteins integral to all cellular membranes and share the defining characteristic of a single transmembrane-domain (TMD) at or near their C terminus (Kutay et al., 1999). As a consequence of this unique structural feature, the TMD of a TA protein emerges from the ribosome only after the termination of translation. Thus, the sorting and insertion of a nascent TA protein are *a priori* post-translational. The TA proteins are therefore distinct from membrane proteins that can also possess a C-terminal TMD, but, in addition, contain another sequence that initiates translocation into the endoplasmic reticulum (ER) via the classical signal recognition particle (SRP)/Sec61 co-translational pathway (Grudnik et al., 2009). The C-terminal TMD of a TA protein also dictates its characteristic membrane orientation, whereby the N-terminal portion of the protein, which often represents the majority of the polypeptide and contains the functional domain(s), faces the cytoplasm, while the C-terminal sequence (CTS) downstream of the TMD, which usually contains organelle-specific targeting information, protrudes into the organelle's interior (Borgese and Fasana, 2011).

The TA proteins are involved in a remarkable array of cellular processes, especially in plants (reviewed in Abell and Mullen, 2011). Some notable examples include the SNAREs (Soluble NSF Attachment protein REceptors), which mediate vesicular transport and fusion (Malsam et al., 2008), subunits of the ER (Osborne et al., 2005), mitochondrial, and plastidial outer membrane translocons (Jarvis et al., 1998; Gutensohn et al., 2000; Werhahn et al., 2001; Allen et al., 2002; Beilharz et al., 2003; Macasev et al., 2004), the electron carrier cytochrome *b*_5_(Cb5) (D'Arrigo et al., 1993; Kuroda et al., 1998; Borgese et al., 2001; Hwang et al., 2004), FIS1 (Fission 1), which is required for organelle fission (Zhang and Hu, 2008; Zhao et al., 2013), and members of the Bcl protein family that are involved in the regulation of apoptosis (Kale et al., 2012). In fact, hundreds of TA proteins have been identified in a wide range of evolutionarily diverse organisms, including humans (Kalbfleisch et al., 2007), *Saccharomyces cerevisiae* (Beilharz et al., 2003), *A. thaliana* (Kriechbaumer et al., 2009; Pedrazzini, 2009; Dhanoa et al., 2010), and bacteria (Borgese and Righi, 2010; Craney et al., 2011), with many of these proteins having unknown function. As such, there is a growing appreciation that TA proteins participate in far more cellular processes than previously envisioned. Moreover, because of their distinct structural characteristics and unusual targeting and membrane insertion pathways, considerable attention has been devoted in recent years to understanding the mechanisms underlying their biogenesis.

By far the most-studied TA proteins in terms of their biogenesis are those localized to the ER, including those that are subsequently transported to other compartments of the endomembrane system, such as the nuclear envelope, Golgi, endosomes, vacuole/lysosomes, plasma membrane, and peroxisomes (reviewed in Rabu et al., 2009; Colombo and Fasana, 2011). For instance, the targeting information responsible for the initial sorting of nascent TA proteins to the ER is well-established as being located within their C termini, including the TMD and CTS (Borgese et al., 2003). The targeting signals for ER-TA proteins are also known not to be based on specific amino acid sequences, but rather consist of general physicochemical properties that are unique to this group of proteins. Compared to mitochondrial-TA proteins, for instance, ER-TA proteins usually contain TMDs that are longer and more hydrophobic, and their CTSs are often less positively charged (Borgese et al., 2003). Indeed, recent structural studies have confirmed that these unique properties in the C termini of ER-TA proteins effectively mediate their specific recognition and insertion by the conserved GET (Guided Entry of TA proteins) and TRC40 (Transmembrane domain Recognition Complex 40) complexes in yeasts and mammals, respectively (reviewed in Denic, 2012). Homologs of the GET3/TRC40 machinery also exist in plants (Abell and Mullen, 2011; Duncan et al., 2013), but their function in ER-TA protein biogenesis has not yet been investigated. There is also the intriguing possibility that the biogenesis of ER-TA proteins in plants involves other, perhaps novel pathways, such as SRP/Sec61 acting in an unusual post-translation mode (Abell et al., 2003) or the ankyrin repeat-containing protein, AKR2A (Shen et al., 2010), which also mediates the targeting of chloroplast outer membrane-(TA) proteins (Bae et al., 2008; Dhanoa et al., 2010).

While the biogenesis of mitochondrial and plastidial-TA proteins is relatively less understood than that of ER-TA proteins, several important points have emerged. For instance, based on the few chloroplast outer membrane TA-proteins studied to date, there appear to be at least two biogenetic pathways, distinguished by the nature of their targeting signals, the membrane protein and lipid components involved (Li and Chen, 1997; Tsai et al., 1999; Qbadou et al., 2003; Dhanoa et al., 2010), and perhaps cytoplasmic components (Kriechbaumer and Abell, 2012). For mitochondrial-TA proteins, targeting to the outer mitochondrial membrane (OMM) is generally considered to be mediated by the distinct physicochemical properties of their C termini. That is, in comparison with the C termini of ER-TA proteins, mitochondrial TA regions usually consist of a shorter and moderately hydrophobic TMD and a positively-charged CTS (Borgese et al., 2003). For most mitochondrial-TA proteins, disruption of either the TMD or CTS results in mislocalization to the ER (D'Arrigo et al., 1993; Mihara, 2000; Borgese et al., 2001; Horie et al., 2002), revealing that these two organelles are linked by independent but competing pathways. There is also mounting evidence that the targeting specificity of mitochondrial-TA proteins, similar to that of plastidial-TA proteins, involves the inherent lipid composition of the membranes in which they properly reside. In yeast, for instance, the relatively low level of ergosterol in the OMM compared to the ER membrane has been shown to play a major role in targeting specificity (Krumpe et al., 2012). In mammals, both membrane lipids (Otera et al., 2007) and membrane protein machinery (Stojanovski et al., 2007) have been implicated in the biogenesis of mitochondrial-TA proteins.

Although no comparable studies have been conducted on whether membrane lipid composition and/or protein machinery serves as a determinant in properly guiding mitochondrial-TA proteins in plant cells, there are data suggesting that the targeting information in plant mitochondrial-TA proteins is more complex than that in yeast and mammalian cells. That is, targeting to the OMM in plants appears to rely on not only similar physicochemical characteristics that are conserved in yeast and mammalian mitochondrial-TA proteins (e.g., short and moderately hydrophobic TMD), but also several discrete sequence-specific features. For instance, in the mitochondrial isoform of cytochrome *b*_5_ from tung tree (i.e., *Aleurites fordii* Hemsl Cb5D), one the most prominent of these features is a dibasic sequence motif in the protein's three-amino-acid-long CTS, whereby the first position of the motif is an arginine, the second position is an arginine, lysine, or histidine and the third position cannot be occupied by a negatively-charged residue (Hwang et al., 2004). Notably, this same dibasic motif (-R-R/K/H-X^{X≠E}^) is present in the CTSs of other putative mitochondrial isoforms of plant Cb5, including *A. thaliana* Cb5 isoform 6 (Cb5-6) (Hwang et al., 2004). The motif is absent, however, in the CTSs of mitochondrial isoforms of mammalian Cb5 (there are no known mitochondrial isoforms of Cb5 in yeast), suggesting that it is an added feature in plant mitochondrial Cb5 proteins that allows them to cope with the need to discriminate between mitochondria, ER and plastids (Hwang et al., 2004; Abell and Mullen, 2011).

Here we show that the C-terminal dibasic motif (-R-R/K/H-X^{X≠E}^) of tung Cb5D exists in several other *A. thaliana* OMM-TA proteins besides Cb5-6, but is absent in most of the TA protein subunits of the TOM (Translocon at the Outer membrane of Mitochondria) complex. We also describe the results of a mutational analysis of selected members from each of these two groups of mitochondrial-TA proteins, indicating that they rely on different types of targeting signals. Moreover, results from mutational analysis of the dibasic targeting signal reveals that this motif is more diverse than previously believed and that the new consensus sequence for this signal not only has promising predictive power for identifying new candidate OMM-TA proteins, but also serves as an important step toward elucidating the differential targeting signals utilized by various classes of TA proteins in plant cells.

## Materials and methods

### Recombinant DNA procedures and reagents

Standard recombinant DNA procedures were performed as described by Sambrook et al. (1989). Molecular biology reagents were purchased from New England BioLabs and Invitrogen. All plasmid DNA constructs were verified using automated dye-terminated cycle sequencing performed at the University of Guelph Genomics Facility. All custom oligonucleotide forward and reverse primers used for polymerase chain reaction (PCR)-based cloning and site-directed mutagenesis (see “*PLASMID CONSTRUCTION*”) were synthesized by Sigma-Aldrich Ltd., and the sequences of these primers are available upon request.

### Plasmid construction

cDNAs encoding full-length open reading frames (ORFs) for the various *A. thaliana* candidate OMM-TA proteins examined in this study were obtained from the *A. thaliana* Biological Resource Center (ABRC) (Ohio State University) or RIKEN Bioresource Center and then, using PCR and the appropriate forward and reverse primers, were sub-cloned as either the entire ORF or, for green fluorescent protein (GFP) fusion proteins consisting of GFP linked to the C terminus of a TA protein, portions thereof, into one or more of the following vectors: pRLT2/Myc-MCS or pRTL2/HA-MCS, plant expression vectors that includes the 35S cauliflower mosaic virus (CMV) promoter and sequences encoding an initiation methionine, glycine linker and Myc or hemagluttinin (HA) epitope tag (Fritze and Anderson, 2000), then a multiple cloning site (MCS) (Shockey et al., 2006); pRTL2/GFP-MCS, which contain the 35S CMV promoter and an MCS immediately 5′ of the GFP ORF (Shockey et al., 2006); or pSPUTK-*Bgl*II, which contains the SP6 promoter, the high-efficiency β-globin 5′ untranslated region, a Kozak's initiation site for efficient translation in rabbit reticulocyte lysate, and an MCS. Complete details on the construction procedures used for generating plasmids encoding any of the various *A. thaliana* TA proteins and all modified versions thereof described in this study are available upon request. pRTL2/Myc-TOM40 encodes the 40 kDa channel-forming subunit of the *A. thaliana* TOM complex fused to an N-terminal Myc epitope tag (Hwang et al., 2008), pRTL2/BCAT3-Cherry encodes the *A. thaliana* plastidial branched-chain aminotransferase 3 fused to the monomeric Cherry fluorescent protein (Niehaus et al., 2014), and pRTL2/Cherry-PTS1 encodes the Cherry protein appended to the C-terminal type 1 peroxisomal matrix targeting signal from pumpkin hydroxypyruvate reductase (Ching et al., 2012).

### By-2 cell cultures, biolistic bombardment, and immunostaining of by-2 cells

*Nicotinana tabacum* Bright Yellow-2 (BY-2) suspension cell cultures were maintained and prepared for bombardment with a biolistic particle delivery system-1000/HE (Bio-Rad Laboratories) as described previously (Lingard et al., 2008). Transient (co-)transformations were performed using 0.5–2 μg of plasmid DNA, which was determined empirically based on the relative strength of the (immuno)fluorescence signal. Bombarded cells were incubated for ~4 h to allow for expression and sorting of the introduced gene product(s). Amounts of plasmid DNA and the ~4 h post-bombardment time point were chosen in order to ensure that any potential negative effects due to excessively high levels of protein expression were diminished. Cells were fixed in 4% (w/v) formaldehyde, followed by permeabilization with 0.01% (w/v) pectolyase Y-23 (Kyowa Chemical Products) and either 0.3% (v/v) Triton X-100 or 25 μg mL^−1^ digitonin (Lee et al., 1997). Primary and dye-conjugated secondary antibodies and sources were as follows: rabbit anti-Myc IgGs (Bethyl Laboratories); mouse anti-Myc antibodies in hybridoma medium (Princeton University, Monoclonal Antibody Facility); mouse anti-α-tubulin (Sigma-Aldrich Ltd.); mouse anti-maize β-ATPase antibodies in hybridoma medium (Luethy et al., 1993) (kindly provided by T. Elthon, University of Nebraska-Lincoln); rabbit anti-cytochrome c oxidase subunit II (CoxII) IgGs (Frelin et al., 2012); goat anti-mouse and goat anti-rabbit Alexa 488 IgGs (Molecular Probes); and goat anti-rabbit and goat anti-mouse rhodamine red-X IgGs (Jackson ImmunoResearch Laboratories). Concanavalin A (ConA) conjugated to Alexa 594 (Molecular Probes) was added to cells at a final concentration of 5 μg/mL during the final 20 min of incubation with secondary antibodies. In all experiments, at least 50 independently transformed cells were evaluated to determine intracellular localization(s) of the transiently-expressed protein, and each biolistic experiment was replicated at least three times.

### *A. thaliana* growth conditions and transformation

All plants were grown in chambers at 21°C with a 16 h/8 h light/dark cycle. Seeds were typically surface sterilized and sown in plant nutrient (PN) media (Haughn and Somerville, 1986) or half-strength Murashige and Skoog (MS) salts (Murashige and Skoog, 1962) containing 0.5% (w/v) sucrose, and solidified with 0.6% (w/v) agar. The stable transgenic line of *A. thaliana* (Columbia-0 ecotype) co-expressing the Cherry-At1g55450 and mito-GFP fusion proteins was generated by transforming plants already expressing mito-GFP, the seeds for which were kindly provided by David Logan (INRA/AgroCampus Ouest/Université d'Angers) (Logan and Leaver, 2000) using the *Agrobacterium tumefaciens* (strain GV3101)-mediated floral dip transformation (Clough and Bent, 1998). Two independent lines co-expressing Cherry-At1g55450/mito-GFP were selected to test for Cherry/GFP fluorescence intensities, and used also for localization studies. None of the transgenic lines displayed any obvious growth or reproductive abnormalities.

### Microscopy

Epifluorescence microscopic images of BY-2 cells were acquired using a Axioscope 2 MOT fluorescence microscope (Carl Zeiss Inc.) equipped with a 63× Plan Apochromat objective and a Retiga 1300 CCD camera (Qimaging), plus associated Openlab software (Improvision). Confocal laser-scanning microscopy (CLSM) images of BY-2 cells and *A. thaliana* 7- to 8-day-old seedlings, which were placed on a glass slide in distilled water under a coverslip and then viewed, were acquired using a Leica DM RBE microscope with a 63× Plan Apochromat objective, TCS SP2 scanning head, and LAS AF software package. CLSM images were acquired as single optical sections of representative cells and were saved as 512 × 512-pixel digital images. All figure compositions and merged images were generated using Openlab (Improvision) or Northern Eclipse (Empix Imaging Inc.) software, and Adobe Photoshop CS (Adobe Systems). All micrographs shown in the figures are representative images obtained in experiments that were replicated at least three times.

### Isolation of insertion-competent mitochondria

Mitochondria were purified from 14-day-old, light-grown pea (*Pisum sativum* cv. Little Marvel) seedlings by a modification of the procedure described by Fang et al. (1987). The seedlings were harvested *en mass* ~2–3 cm above the soil line, using a large pair of scissors. All subsequent steps were on ice or at 4°C. Plant material was rinsed with deionized water, then homogenized at 1 g fw/2.5 mL of homogenization medium using 3–10 s bursts with a Braun blender modified to hold single-edged razor blades. Homogenization medium was 40 mM MOPS-KOH, pH 7.2, containing 600 mM mannitol, 10 mM EDTA, 8 mM cysteine (free base), and 0.4% defatted BSA. The homogenates were filtered through 8 layers of cheesecloth plus 2 layers of Miracloth (Fisher Scientific). The filtrates were centrifuged at 3300 × g for 5 min, and the 3.3 k pellets were discarded. The supernatants were centrifuged at 18,000 × g for 20 min, and the supernatants discarded. The 18 k pellets were resuspended in 35 mL of 5 mM MOPS-KOH, pH 7.2, containing 250 mM mannitol, and 0.1% defatted BSA, first by using a horse-hair paint brush, then by 3 passes in a loose-fitting glass and Teflon Potter-Elvehjem homogenizer. Eight mL of the resuspended mitochondria-enriched fraction was layered on top of a discontinuous Percoll step gradient comprised of 6 mL 21% (v/v), 12 mL 26%, 10 mL 47%, all in 5 mM MOPS-KOH, pH 7.2, containing 250 mM mannitol. The Percoll gradients were centrifuged at 65,000 × g for 45 min using a Beckman SW-28 rotor. The mitochondria, which band at the 26/47% Percoll interface, were collected, diluted with 10 volumes of 5 mM MOPS-KOH, pH 7.2, containing 250 mM mannitol and 10 mM DTT, and centrifuged at 18,000 × g for 15 min. The 18 k pellet was resuspended in MOPS/mannitol/DTT and re-pelleted. The final mitochondria-enriched pellets were resuspended in a small volume of MOPS/mannitol/DTT, and kept on ice prior to import experiments.

### Plasmid-driven *in vitro* transcription/tranlsation/insertion

The *in vitro* transcription and translation reactions were conducted while the mitochondria were being prepared. Unless otherwise noted, all reagents were from Sigma Chemical Co. Purified, linearized plasmids (pSPUTK/Myc-At1g55450 and pSPUTK/Myc-TraB) were used for T7-driven coupled transcription-translation in a rabbit reticulocyte lysate (Promega) plus EasyTag L-[35S]-Met (Perkin Elmer) according to the manufacturer's instructions (Promega). Translations were terminated after 90 min at 30°C by adding 100 μg emetine, and ribosomes were removed by centrifugation at 150,000 × g for 15 min at 4°C using a Beckman Model TLA 100.2 rotor. Unincorporated [35S]-Met was removed from the supernatant using Centri-spin 10 desalting columns (Princeton Separations).

Import/integration reactions were conducted in 200 μL total volume, contained 100 μg mitochondrial protein, and typically 1–5% (v/v) of the translation reaction. Mitochondria were diluted into 25°C integration buffer and pre-incubated for 5 min prior to adding the 35S-labeled translation products. Integration buffer consisted of 300 mM sorbitol, 10 mM HEPES-KOH, pH 7.2, 0.1% defatted-BSA, 80 mM KCl, 10 mM MgOAc, 2 mM KH_2_PO_4_, and 1 mM MgCl_2_. Reactions using energized mitochondria additionally contained 2 mM L-malate, 4 mM NADH, 2 mM ATP, pH 7.0, 60 mM phospho-creatine, and 0.15 mg/mL creatine kinase. Integration reactions were incubated at 25°C for 20–25 min. For protein import, reaction mixtures were then transferred to ice and incubated for 20 min with 10 μg/mL Proteinase K. To stop proteolysis, PMSF was added to 2 mM. Membrane integration was determined essentially as described (Fujiki et al., 1982). Following a wash with 100 mM Na_2_CO_3_, pH 11.5 (ASC), membranes were pelleted by centrifugation at 150,000 × g for 30 min at 4°C using a Beckman Model TLA 100.2 rotor. Pellets were washed one time by resuspending in ASC and re-pelleting. 35S-translation products were acid precipitated and washed by resuspensing then re-pelleting. All samples were analyzed by SDS-PAGE plus phosphor-imaging. Equal volumes of sample and 8 M urea, 4% SDS, and 4% 2-mercaptoethanol were combined, heated to 60°C for 5 min, and clarified by centrifugation at maximum speed in a micro-centrifuge for 1 min. Proteins were separated after application to pre-cast Novex NuPAGE 12% Bis-Tris gels using the MES/SDS buffer (Life Technologies). Electrophoresis was stopped when the dye-front reached the bottom of the gels. After drying, gels were wrapped in cellophane, placed onto a K-type phosphor-imaging screen, and analyzed for 16–18 h at room temp using a Bio-Rad PMI system.

### Bioinformatics analysis

A list of all putative TA proteins in *A. thaliana* was compiled from previously published TA protein datasets (Kriechbaumer et al., 2009; Pedrazzini, 2009), plus those identified using the “TAMP (TA Membrane Protein) Finder” program (Dhanoa et al., 2010; Craney et al., 2011). Additional information on the TAMP Finder program and the putative *A. thaliana* TA proteins identified using this program will be published elsewhere. Known and putative *A. thaliana* OMM-TA proteins that contain an expanded dibasic motif in their CTS, as defined in this study, e.g., -R/K/H-X^{0,1≠E}^-R/K/H^{≠−H−H−or−H−X−H−}^-X^{0,1≠E}^X^{0,3}{CTS=3,8}^ (**Table 2**), were identified initially by visual inspection of the Kriechbaumer et al. (2009), Pedrazzini (2009), and TAMP datasets. However, since there is no established computational method for precisely defining the ends of a TMD and thus, the length of the CTS, all proteins containing the motif within a CTS predicted to be 2 to 10 amino acids in length were considered candidates. The TMD predictions were performed using TMPred (Hofmann and Stoffel, 1993), ARAMEMNON (Schwacke et al., 2003), and/or TOPCONS (Bernsel et al., 2009). Candidate proteins were analyzed using the TargetP 1.1 Server (http://www.cbs.dtu.dk/services/TargetP/) (Emanuelsson et al., 2000) and PEPscreen® Calculator (Sigma-Aldrich Ltd; according to Monera et al., 1995) or Grand Average of Hydropathy (GRAVY) Calculator (http://www.gravy-calculator.de/) and those proteins predicted to contain an N-terminal targeting signal (based on a >0.51 cutoff for TargetP) for mitochondria, chloroplasts or the ER (secretory pathway) and/or a predicted TMD with a relatively high hydropathy score (based on a >1.2 cutoff for PEPscreen®) were excluded. Thereafter, duplicated proteins due to splice variants were removed. All deduced amino acid sequences were obtained from GenBank and/or The *Arabidopsis* Information Resource (TAIR). Predicted intracellular localizations were taken from SUBA3 (The SUBcellular localization database for *A. thaliana* proteins) (Tanz et al., 2013) or the Gene Ontology database (Ashburner et al., 2000), or were experimentally determined here.

## Results and dicussion

### Identification of two major groups of OMM-TA proteins in *a. thaliana*

To begin to further analyze the targeting signals in mitochondrial-TA proteins in plants, we first compiled a list of *bona fide* OMM proteins that are also considered to have a TA-orientation. Specifically, we cross-referenced datasets of authentic *A. thaliana* mitochondrial membrane proteins previously identified in various proteomics screens (Duncan et al., 2011; Klodmann et al., 2011; reviewed in Duncan et al., 2013) with all of the TA proteins predicted for the *A. thaliana* deduced proteome (Kriechbaumer et al., 2009; Pedrazzini, 2009; Dhanoa et al., 2010). As shown in Table 1, a total of 20 candidate mitochondrial TA-proteins were identified, including 10 subunits of the TOM complex, as well as FIS1A/B and PMD1/2 (Peroxisomal and Mitochondrial Division factor 1 and 2), which serve together as regulators of mitochondrial fission (Scott et al., 2006; Zhang and Hu, 2009; Aung and Hu, 2011), and MIRO1/2 (MItochondrial RHO GTPases 1 and 2), which regulate mitochondrial morphology and motility (Yamaoka and Leaver, 2008). Also identified were isoforms of ascorbate peroxidase (APX-5) (Caverzan et al., 2012) and purple acid phosphatase (PAP2) (Sun et al., 2012), as well as a member of the TraB protein family (At1g05270), which, based on the role of its homologs in bacteria, is thought to play a role in signaling in plants (Duncan et al., 2011). Also listed in Table 1, as expected, is Cb5-6, which, as already mentioned, is one of six isoforms of Cb5 in *A. thaliana* (Maggio et al., 2007; Paquette et al., 2009) that was experimentally determined to possess a similar OMM dibasic targeting signal motif as that found in tung mitochondrial Cb5D (Hwang et al., 2004).

**Table 1:**
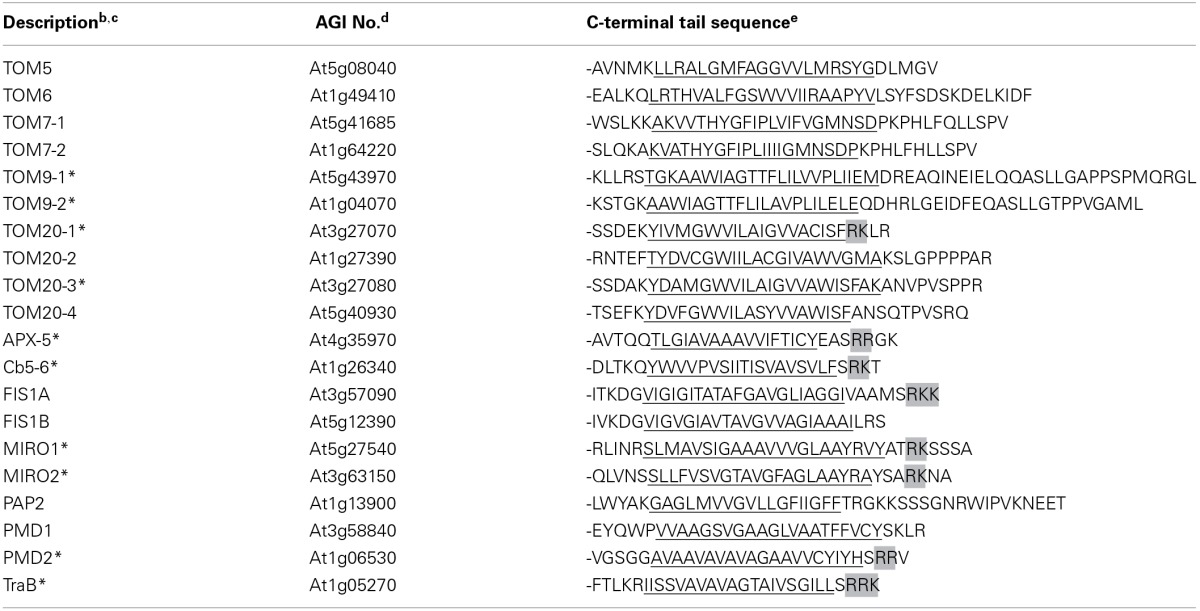
***Arabidopsis* OMM TA proteins^a^**.

a All of the proteins listed are bona fide Arabidopsis OMM proteins, based on (Duncan et al., 2011, 2013) and Klodmann et al. (2011), and are also known or predicted to possess a TA topology, based on their identification in various proteomics- and bioinformatics-based searches in Arabidopsis (Kriechbaumer et al., 2009; Pedrazzini, 2009; Dhanoa et al., 2010). The proteins are grouped by TOM proteins and all other proteins, and then listed alphabetically. See text for additional details.

^b^Common nomenclature of Arabidopsis mitochondrial outer membrane TA proteins based on published literature and The Arabidopsis Information Resource (TAIR) (http://www.arabidopsis.org). Proteins indicated with an asterisk were experimentally characterized in terms of their intracellular localization and TA topology; see **Figures 1, 2**, and text for additional details.

^c^Abbreviations are: APX-5, ascorbate peroxidase isoform 5; Cb5-6, cytochrome b_5_ isoform 6; FIS1, fission 1; MIRO1/2, mitochondrial RHO GTPase 1 and 2; PAP2, purple acid phosphatase 2; PMD1/2, peroxisomal and mitochondrial division factor 1 and 2; TOM, translocase of the mitochondrial outer membrane (subunits 5, 6, 7, 9, and 20 and isoforms thereof); TraB, domain motif based on Enterococcus faecalis traB (An and Clewell, 1994).

^d^Arabidopsis gene identifier (AGI) number represents the systematic designation given to each locus, gene, and its corresponding protein product(s) by TAIR.

^e^Shown for each protein is its deduced C-terminal tail sequence, including its putative TMD (underlined), based on the TMD prediction program TOPCONS and visual inspection, and its downstream CTS. Shaded are the dibasic amino acid residues in the dibasic targeting signal motif, -R-R/K/H-X^{X≠E}^ (Hwang et al., 2004).

Inspection of the CTSs presented in Table 1 shows that while all of the proteins possess a relatively short (<21 residues) and moderately hydrophobic TMD, there is a clear separation of the proteins into two groups (*p* = 0.009, hypergeometric test) based on their CTSs; (i) the TOM proteins, which, with the exception of TOM20-1, generally have longer CTSs that lack a dibasic motif (i.e., -R-R/K/H-X^{X≠E}^), and (ii) all other proteins, the majority of which have relatively shorter CTSs and contain a dibasic motif. Notably, several of the proteins in the dibasic group, including Cb5-6, FIS1A/B, PAP2, and PMD2 have been reported to target not only to mitochondria, but also to other organelles (i.e., peroxisomes or chloroplasts) in certain plant cell types and/or experimental conditions (Scott et al., 2006; Maggio et al., 2007; Lingard et al., 2008; Zhang and Hu, 2009; Aung and Hu, 2011; Sun et al., 2012; Ruberti et al., 2014). Two of these proteins contain what appear to be more divergent CTSs than the other proteins in this group, including FIS1B, which contains only a single basic amino acid in its CTS (i.e., -LRS), and PAP2, which has a relatively long CTS (i.e., 20 amino acids) and also contains several basic residues, including a dilysine sequence, and acidic residues. Perhaps some of these sequence differences in the CTSs, as well as others in the TMD and/or additional factors, contribute to their alternative subcellular localizations. For instance, binding of another protein, including a chaperone and/or receptor that itself might vary depending on cell type and/or environmental condition, burying/exposing of the targeting elements by protein folding or, as suggested previously for Cb5 (Maggio et al., 2007), post-translational modification (e.g., phosphorylation), may be also involved in the differential targeting of these proteins. Clearly, further work is needed to better understand how these OMM-TA proteins discriminate between different organelles in plant cells.

Taken together, these results indicate that many OMM-TA proteins in *A. thaliana* contain a dibasic motif similar to that known to be important for the targeting of mitochondrial Cb5 from tung (Hwang et al., 2004). Furthermore, the absence of this dibasic motif in the CTSs of most of the TOM-TA proteins suggests that these proteins rely on a different type of targeting signal for their localization to the OMM.

### Localization and topology of OMM TA proteins in tobacco by-2 cells

**Figure 1:**
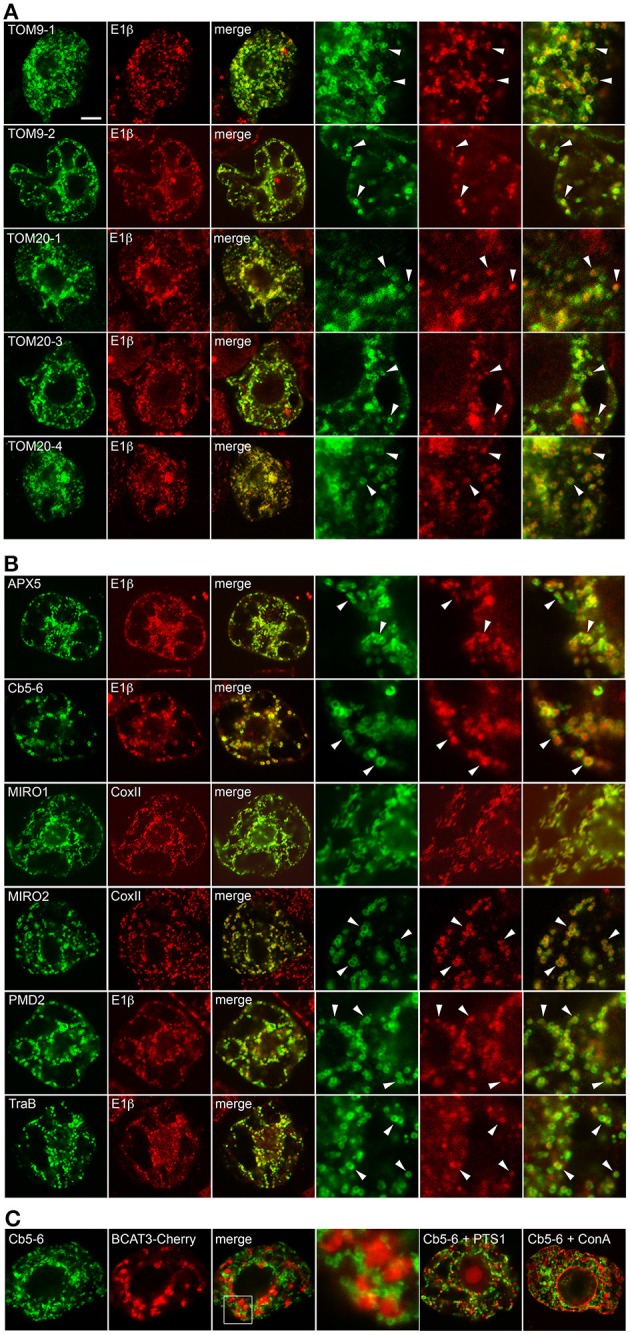
**Subcellular localization of selected *A. thaliana* OMM-TA proteins in BY-2 cells**. BY-2 cells were transiently transformed with plasmid DNA expressing selected Myc-tagged TOM-TA proteins **(A)** or dibasic-motif-containing TA proteins **(B)** and immunostained for endogenous mitochondrial E1β or CoxII, as indicated in the panel labels. Alternatively, in **(C)**, cells were (co-)transformed with Myc-tagged Cb5-6 and the plastid marker protein BCAT3-Cherry or the peroxisome marker protein (PTS1), which also includes the Cherry protein, or incubated with fluor-conjugated concanavalin A (ConA), serving as an ER marker stain (Tartakoff and Vassalli, 1983). Processing of cells for immunofluorescence microscopy and viewing using CLSM are as described in the “Materials and Methods.” Shown in the three panels on the right side of each row in **(A)** and **(B)** are images corresponding to a portion of the cell at higher magnification. Solid arrowheads indicate examples of the torus-shaped fluorescent structures containing the Myc-tagged TA protein delineating the spherical structures attributable to mitochondrial E1β or CoxII. The box in **(C)** represents the portion of the Cb5-6 and BCAT3-Cherry co-transformed cells shown at higher magnification in the panel to the right. Note also in **(C)** that only merged images of a Cb5-6 and PTS1 co-transformed cell or a Cb5-6-transformed cell stained with ConA are shown. Bar in **(A)** = 10 μm.

To analyze the targeting information present in the two groups of proteins presented in Table 1, we first confirmed the intracellular localization of a subset of proteins from each group. Plasmid DNAs encoding the respective proteins were transiently expressed and visualized in tobacco BY-2 suspension-cultured cells, which are commonly used as model system for studying protein localization and targeting (Brandizzi et al., 2003; Miao and Jiang, 2007; Denecke et al., 2012). As shown in Figures 1A,B, CLSM images of BY-2 cells transiently-expressing N-terminal Myc-epitope-tagged members from both groups of proteins revealed that all of the proteins localized to mitochondria. That is, all of the proteins, with the exception of MIRO1, displayed a torus or ring-shaped immunofluorescence pattern that encircled the punctate immunofluorescence pattern attributable to the endogenous mitochondrial pyruvate dehydrogenase E1β (Luethy et al., 1993) or CoxII (Millar et al., 2011) (Figures 1A,B). Transiently-expressed MIRO1 also targeted to mitochondria, but appeared to alter the morphology of the organelle (Figure 1B), similar to what is observed for its mammalian and yeast protein counterparts (Fransson et al., 2003; Frederick et al., 2004). Similar localization results were reported for several of the same proteins when they were transiently expressed as GFP-tagged proteins in *A. thaliana* suspension cells (Duncan et al., 2011), indicating that the appended tag (Myc or GFP) does not influence intracellular localization. Notably, we also demonstrated that the mitochondrial localization of the proteins examined in this study in BY-2 cells was readily distinguishable from various other organelles, namely plastids (i.e., leucoplasts), peroxisomes, and the ER [results presented for Cb5-6 (Figure 1C)].

**Figure 2:**
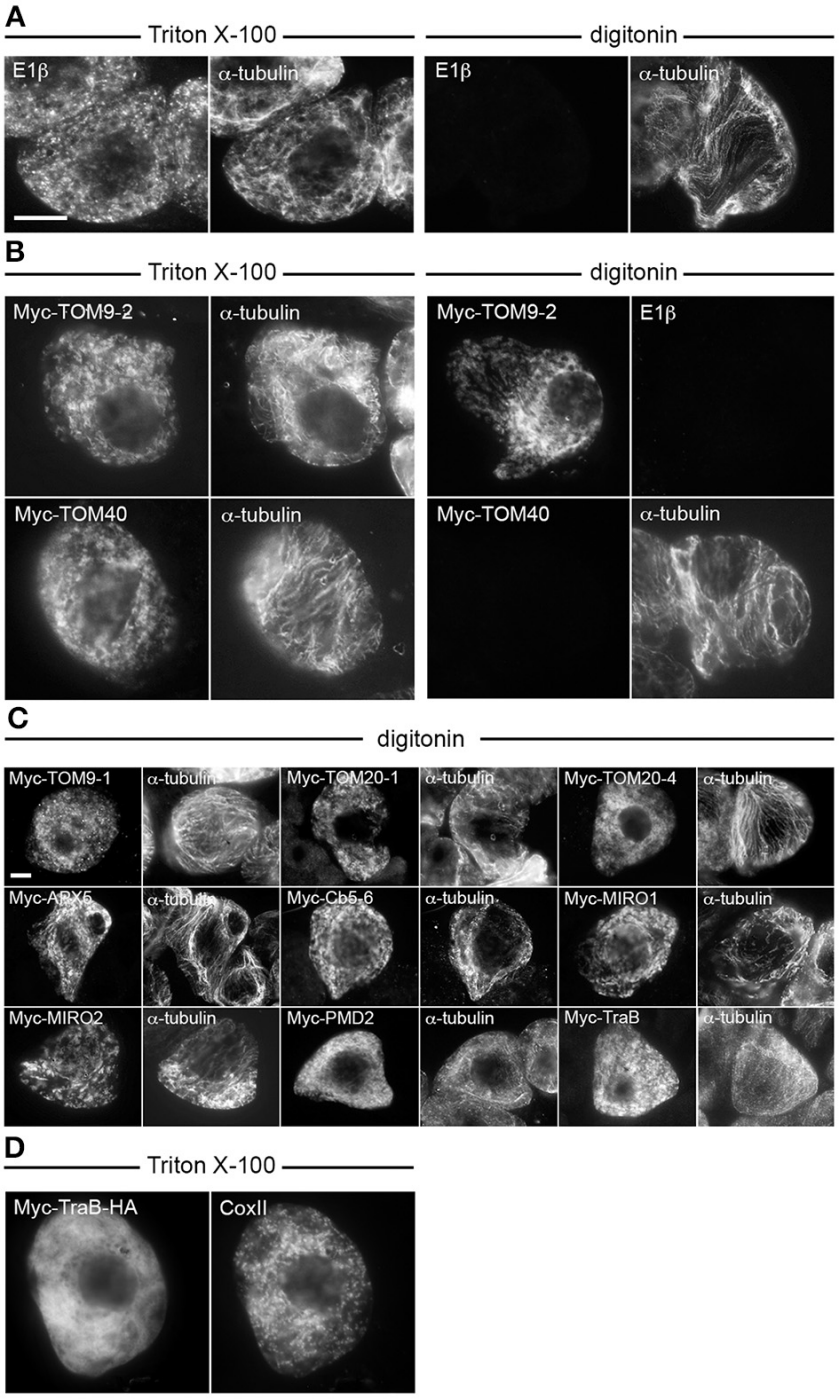
**Topological mapping of selected *A. thaliana* OMM-TA proteins in differentially permeabilized BY-2 cells**. Non-transformed **(A)** or transiently-transformed **(B–D)** BY-2 cells were formaldehyde fixed and permeabilized (as indicated above each set of images) with either Triton X-100, which perforates all cellular membranes, or, digitonin, which selectively permeabilizes the plasma membrane, then cells were processed for immuno-epifluorescence microscopy. Also indicated in each panel is the name of the immunostained transiently-expressed Myc-tagged protein or endogenous protein (i.e., E1β, CoxII or α-tubulin). Note that the presence or absence of immunofluorescence reflects whether the protein (epitope) was accessible to the applied antibodies. For instance, similar to α-tubulin in cytoplasmic microtubules **(A)**, N-terminal Myc-tagged TOM9-2 **(B)** and all other known or putative TA proteins examined **(C)**, but not endogenous E1β in the mitochondrial matrix or the control protein, Myc-TOM40 **(B)**, were immunodetected in digitonin-permeabilized cells. Note also in **(D)** that Myc-TraB-HA did not colocalize with endogenous mitochondrial CoxII, indicating that the expressed protein, unlike Myc-TraB (Figure 1) is not properly targeted to mitochondria. Bar in **(A,C)** = 10 μm.

We next determined whether the selected proteins from Table 1 adopt a TA orientation in mitochondria, e.g., N_cytoplasm_-C_inner membrane space_, by performing differential detergent permeabilization experiments. Permeabilization of BY-2 cells with Triton X-100 solubilizes all cellular membranes, and thus applied antibodies can access epitopes present on proteins inside organelles (e.g., mitochondrial matrix pyruvate dehydrogenase E1β) or in the cytoplasm (e.g., α-tubulin) (Figure 2A). Permeabilization of BY-2 cells with digitonin, however, results in solubilization of only the plasma membrane, and thus applied antibodies can access only epitopes present in the cytoplasm (Figure 2A). As shown in Figure 2B, differential permeabilization of BY-2 cells transiently-expressing N-terminal-Myc-tagged TOM9-2 (Myc-TOM9-2), which is known to be oriented with its N terminus in the cytoplasm (Macasev et al., 2004; Hwang et al., 2008; Carrie et al., 2010) resulted in immunodetection of the protein in cells permeabilized with either Triton X-100 or digitonin, confirming the expected orientation of the protein's N terminus. By contrast, differential permeabilization of BY-2 cells expressing Myc-TOM40, which is a pore-forming subunit of the TOM complex (Macasev et al., 2004; Carrie et al., 2010) and known to be orientated in the OMM such that both its N and C termini face the inner membrane space (Hwang et al., 2008), prevented immunodetection of the protein in BY-2 cells that were permeablized with digitonin, as also expected (Figure 2B).

Shown in Figure 2C are the results of differential permeabilization experiments indicating that, like Myc-TOM9-2, all of the selected Myc-tagged proteins described in Figure 1 were immunodetected in digitonin-permeabilized BY-2 cells (Figure 2C), indicating that they are also orientated in the OMM such that their N termini are exposed to the cytoplasm. We did not test, however, any C-terminal-epitope-tagged-versions of these proteins (or Myc-TOM9-2), since addition of an epitope tag or fluorescent protein to the C terminus of a TA protein, particularly mitochondrial-TA proteins, usually disrupts proper targeting (Borgese et al., 2003; Borgese and Fasana, 2011). Indeed, consistent with the mislocalization of the mitochondrial isoform of mammalian Cb5 with a C-terminal-appended epitope containing an N-glycosylation sequence (Maggio et al., 2007), we observed that Myc-tagged TraB with an added C-terminal-appended HA epitope tag (Myc-TraB-HA) did not properly target to mitochondria in BY-2 cells (Figure 2D). Nonetheless, the data presented Figures 1, 2 indicate that (i) all of the selected proteins from Table 1 target to the OMM when transiently expressed in tobacco BY-2 cells, (ii) these proteins likely adopt the expected TA topology, and (iii) tobacco BY-2 cells can serve as a useful model system to further characterize the targeting signals involved in mitochondrial localization.

### The TA regions of dibasic motif-containing proteins are both necessary and sufficient for targeting to mitochondria, while the TA regions of TOM TA proteins are necessary, but not sufficient for targeting

**Figure 3:**
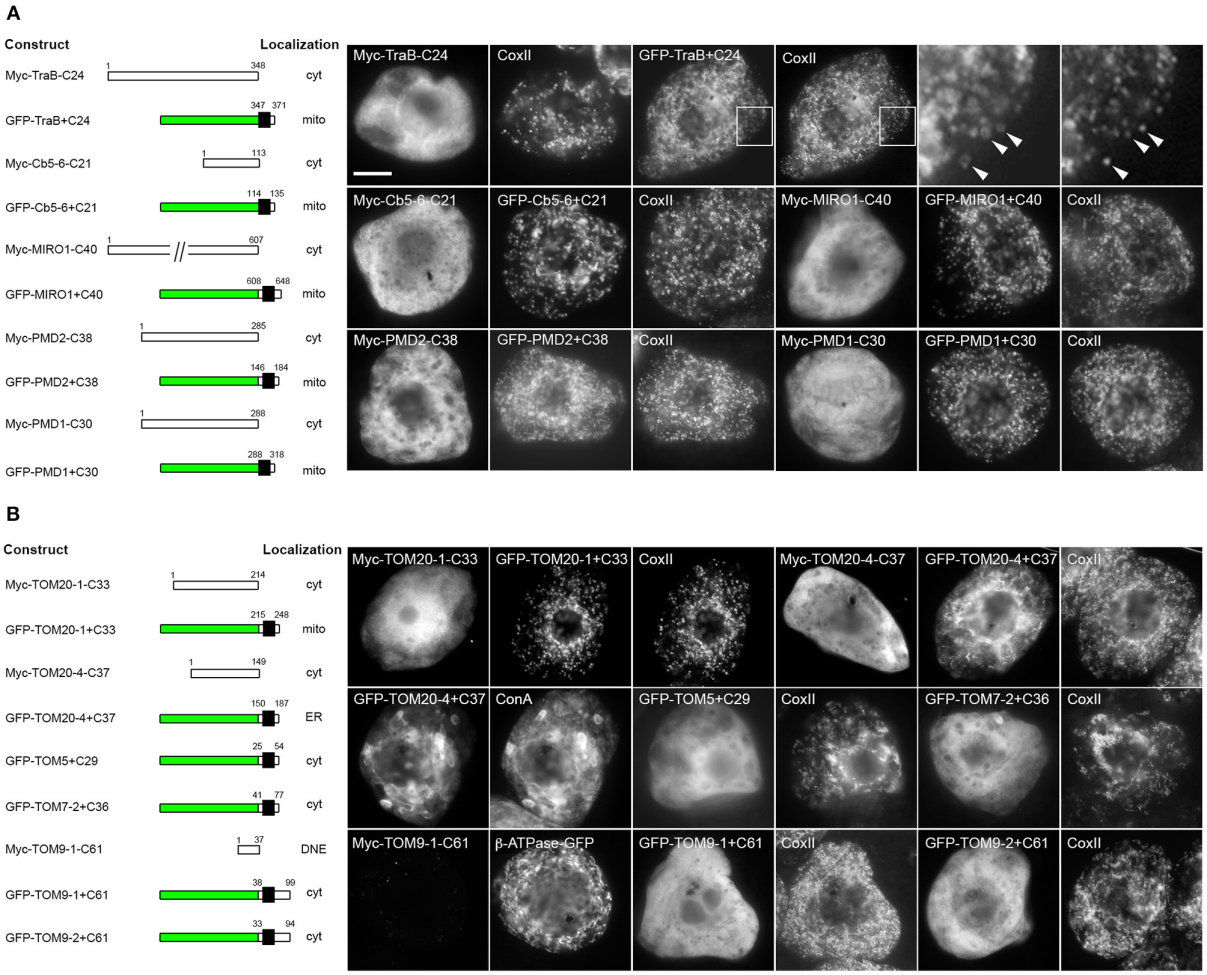
**Localization of various C-terminal mutant and GFP fusions of selected mitochondrial-TA proteins in BY-2 cells**. Shown on the left in both **(A)** and **(B)** are schematic illustrations of various C-terminal-mutant (i.e., truncated) versions or GFP fusions of various dibasic-motif-containing TA proteins **(A)** or TOM-TA proteins **(B)** and their corresponding intracellular localization in transformed BY-2 cells. The numbers in the name of each construct denote the number of residues that were either deleted from the C terminus of the Myc-tagged wild-type TA protein or fused to the C terminus of GFP, and the numbers above each illustration correspond to the N- and C-terminal amino acid residues of the TA protein. Portions of the TA protein are represented in the illustrations by white and black boxes, the latter denoting the putative TMD; green boxes denote GFP. Cyt, cytoplasm; DNE, did not express; ER, endoplasmic reticulum; mito, mitochondria. Shown on the right in both **(A)** and **(B)** are representative immuno-epifluorescence micrographs illustrating the localization of the various constructs shown on the left. Each micrograph is labeled with the name of either the transiently-expressed Myc-tagged C-terminal mutant or GFP fusion protein, the endogenous mitochondrial marker protein, CoxII, or ConA. Boxes in the top row of **(A)** represent the portions of cells shown at higher magnification in the panels to the right. Arrowheads indicate examples of the torus-shaped fluorescent structures containing GFP-TraB+C24 delineating the spherical structures attributable to matrix-localized CoxII, indicating that GFP-TraB+C24 localizes to the OMM. For all other expressed proteins, only general (i.e., lower magnification) fluorescence patterns were compared with those of mitochondrial CoxII or, in the case of GFP-TOM20-4+C37, ConA-stained ER. Note also that cells transformed with Myc-TOM9-1-C61, which did not display a detectable immunofluorescence signal, were identified based on the fluorescence attributable to co-expressed β-ATPase-GFP, serving as cell transformation and mitochondrial matrix marker protein. Bar in **(A)** = 10 μm.

To begin to characterize the targeting information in the two groups of OMM-TA proteins, we first tested whether their C-terminal TA regions, which include both the TMD and CTS, were necessary and/or sufficient for proper localization. Toward this end, we focused first on the group of proteins that contains the dibasic motif, or a divergent version thereof. As shown in Table 1, the CTS of TraB (i.e., -SRRK) closely matches the dibasic motif defined previously for mitochondrial Cb5, that is, -R-R/K/H-X^{X≠E}^. Similar to previous studies of Cb5 (Hwang et al., 2004), the C-terminal TA region of TraB was both necessary and sufficient for localization to mitochondria. As shown in Figure 3A, a mutant version of Myc-tagged TraB lacking its C-terminal 24 amino acid TA region (i.e., Myc-TraB-C24), was localized to the cytoplasm in BY-2 cells and not to CoxII-containing mitochondria, indicating that the TA region is necessary for its proper targeting. Addition of the TraB 24-amino-acid-long TA region to the C terminus of GFP (GFP-TraB+C24), on the other hand, resulted in mitochondrial localization (Figure 3A), indicating that the region is sufficient for targeting. Similarly, the TA sequences of Cb5-6, MIRO1, PMD2, as well as PMD1, the latter of which contains a more divergent dibasic motif in its CTS that harbors a single amino acid insertion between two basic residues (i.e., -SKLR), were all necessary and sufficient for mitochondrial localization (Figure 3A). Given the differences in the TMDs and CTSs of these proteins (Table 1), it appears that a certain degree of sequence divergence, including positioning of the dibasic motif relative to the end of the TMD and/or C terminus, the length of the CTS, and, with respect to PMD1, the contiguous nature of the dibasic sequence itself, can be tolerated while maintaining proper localization to mitochondria.

In contrast to the mitochondrial-TA proteins that contain a dibasic motif in their CTS, the TA regions of TOM proteins were necessary, but not sufficient for mitochondrial localization (Figure 3B). That is, with one exception, all of the TOM-TA proteins were mislocalized to the cytoplasm [or did not express, possibly due to instability/degradation of the truncated protein (i.e., TOM9-1-C61)] when their TMD and CTS were removed, while the addition of these sequences to the C terminus of GFP resulted in localization of the fusion proteins to either to the cytoplasm or, in the case of GFP-TOM20-4+C37, to the ER (Figure 3B). The one exception was TOM20-1, where the TA region was both necessary and sufficient for mitochondrial targeting (Figure 3B). Interestingly, TOM20-1 is distinct from the other TOM TA proteins in that its CTS is relatively short and includes a dibasic sequence similar to the other group of proteins (Table 1). Overall, these data indicate that the targeting information in the two groups of mitochondrial-TA proteins is essentially different, and that for most of the TOM-TA proteins, sequences in addition to their TA regions are required for proper targeting to mitochondria.

### The CTSs of both groups of mitochondrial-TA proteins are necessary for targeting, but only those containing a dibasic motif are interchangeable

The CTSs of most TA proteins contain information that, along with their TMD, is important for ensuring that they are targeted to the proper intracellular membrane (Borgese and Fasana, 2011; Abell and Mullen, 2011). As such, removal of the CTS from the TA region or swapping the CTS with the CTS of another TA protein that localizes to a different organelle usually results in mislocalization. On the other hand, swapping of the CTSs of TA proteins that localize to the same organelle sometimes preserves targeting, at least in those cases where the proteins contain a conserved targeting signal(s).

**Figure 4:**
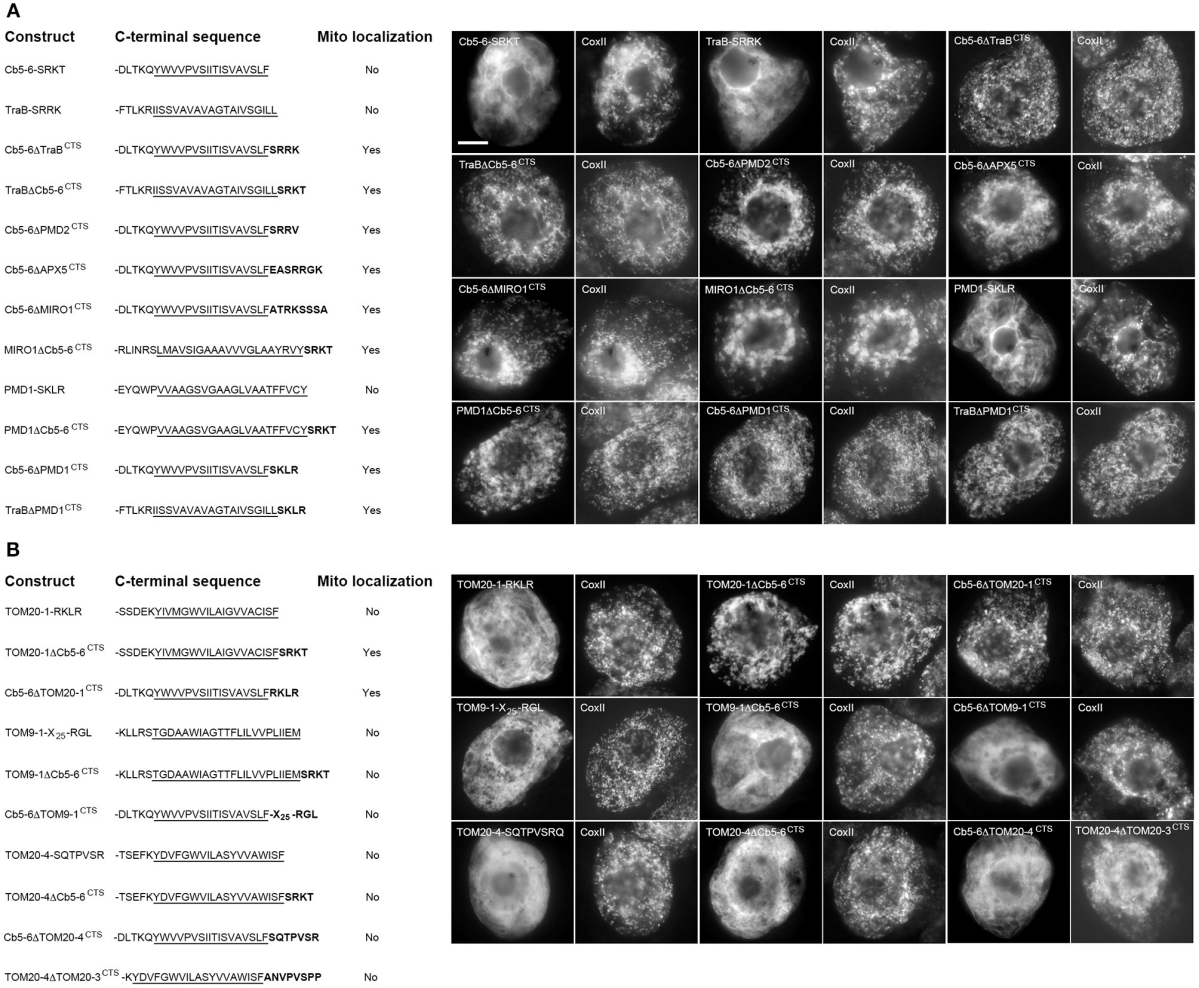
**Localization of various CTS mutants and hybrid versions of selected mitochondrial-TA proteins in BY-2 cells**. Shown on the left in both **(A)** and **(B)** are schematic illustrations of various CTS mutant (truncated) or hybrid versions of selected dibasic-motif-containing TA proteins **(A)** and/or TOM-TA proteins **(B)** and their corresponding localization (or lack thereof) to mitochondria in transformed BY-2 cells. The names of the mutant and hybrid constructs represent either the specific amino acids in the CTS that were deleted from the protein or replaced with the CTS from another protein. All constructs possess an N-terminal-appended Myc-epitope tag. Shown for each construct is the corresponding C-terminal amino acid sequence, including putative TMD (underlined) and modified CTS (bolded), or lack thereof. Mitochondrial localization (indicated as “Yes” or “No”) was assessed based on colocalization (or lack thereof) of the expressed protein and the endogenous mitochondrial CoxII. Shown on the right in both **(A)** and **(B)** are representative immuno-epifluorescence micrographs illustrating the localization of the various constructs shown on the left. Each micrograph is labeled with the name of either the expressed Myc-tagged CTS mutant or hybrid protein or endogenous CoxII. Bar in **(A)** = 10 μm.

To test whether the CTSs of mitochondrial-TA proteins that possess a dibasic motif are interchangeable, a series of truncation and chimeric mutants were generated and their localization was assessed in transiently-transformed BY-2 cells as either mitochondrial or not mitochondrial based on comparison to endogenous CoxII immunostaining in the same cells (Figure 4A). Deletion of the CTS from either Cb5-6 (Cb5-6-SRKT) or TraB (TraB-SRRK) disrupted their localization to mitochondria, indicating that the CTSs of these two proteins, similar to tung Cb5D (Hwang et al., 2004), contain essential mitochondrial targeting information. On the other hand, chimeric versions of Cb5-6 and TraB, whereby their CTSs were swapped, i.e., Cb5-6ΔTraB^CTS^ and TraBΔCb5-6^CTS^, localized to mitochondria (Figure 4A). Similarly, the targeting of Cb5-6 to mitochondria was preserved when its CTS was replaced with the CTS from either PMD2, APX-5, or MIRO1 (Figure 4A), revealing that a CTS containing an acidic amino acid (i.e., glutamic acid in APX-5 [-EASRRGK]), as well as a longer CTS (i.e., 8 amino acids in MIRO1 [-ATRKSSSA]), is acceptable for targeting to mitochondria in a chimeric context (Figure 4A). Likewise, replacement of the longer CTS of MIRO1 with the shorter CTS of Cb5-6 (MIRO1ΔCb5-6^CTS^) preserved mitochondrial localization (Figure 4A). As shown also in Figure 4A, deletion of the CTS from PMD1, which contains a more divergent dibasic motif harboring a single amino acid insertion between two basic residues (i.e., -SKLR), disrupted its mitochondrial localization, yet chimeric versions of PMD1 and either Cb5-6 (PMD1ΔCb5-6^CTS^, Cb5-6ΔPMD1^CTS^) or TraB (TraBΔPMD1^CTS^) localized to mitochondria (Figure 4A). Collectively, these results indicate that not only are the CTSs of TA proteins that include a dibasic motif functionally interchangeable in terms of mitochondrial targeting, but that the dibasic motif can be positioned within CTSs of various lengths. Moreover, these results expand the previous definition of the dibasic motif as -R-R/K/H-X^{X≠E}^ (Hwang et al., 2004), revealing that a dibasic-containing CTS can tolerate a negatively-charged residue, albeit if located further upstream, and that the dibasic amino acids do not have to be contiguous.

We also examined a similar series of CTS truncation and chimeric TOM-TA proteins. As shown in Figure 4B, deletion of the CTS from TOM20-1, which, unlike the other TOM-TA proteins, contains a dibasic motif in its CTS (i.e., -RKLR), resulted in mislocalization. However, addition of the Cb5-6 CTS to this TOM20-1 mutant (TOM20-1ΔCb5-6^CTS^) restored mitochondrial localization (Figure 4B). Similarly, a chimera consisting of Cb5-6 with its CTS replaced with the CTS of TOM20-1 (Cb5-6ΔTOM20-1^CTS^) localized to mitochondria (Figure 4B), indicating that the TOM20-1 CTS is functionally interchangeable with the CTSs from other (non-TOM) TA proteins that possess a dibasic motif. The CTSs of other TOM-TA proteins, however, were not interchangeable with the CTS of Cb5-6. For instance, deletion of the 28 amino-acid-long CTS of TOM9-1 resulted in the modified protein (TOM9-1-X_25_-RGL) being mislocalized, which was still the case when the Cb5-6 CTS was appended to this mutant (TOM9-1ΔCb5-6^CTS^) (Figure 4B). Furthermore, the longer CTS of TOM9-1 could not functionally replace the shorter, dibasic-motif-containing CTS of Cb5-6, i.e., Cb5-6ΔTOM9-1^CTS^ was not localized to mitochondria (Figure 4B). Similarly, deletion of the CTS from TOM20-4 (i.e., TOM20-4-SQTPVSR), which has a significantly shorter CTS than TOM9-1 [i.e., 10 vs. 28 amino acid residues (Table 1)], abolished mitochondrial targeting. Moreover, the CTS of TOM20-4 CTS and Cb5-6 could not be exchanged, since both TOM20-4ΔCb5-6^CTS^ and Cb5-6ΔTOM20-4^CTS^ were mislocalized (Figure 4B). In fact, even replacement of the TOM20-4 CTS with the similar CTS of TOM20-3 (Table 1) resulted in the chimeric protein (TOM20-4ΔTOM203^CTS^) being mislocalized (Figure 4B), revealing that the CTSs of even two closely related TOM proteins (isoforms) could not be exchanged without disrupting proper targeting to mitochondria.

Collectively, the results for the TOM proteins indicate that while their CTSs are necessary for targeting, they likely require other unique sequences within the context of the TMD and/or elsewhere in the native protein to function properly. This conclusion is perhaps not surprising given that the targeting of at least some TOM-TA proteins in other organisms has been shown to rely on unique sequences upstream of their CTSs, including for *S. cerevisiae* TOM22 (Rodriguez-Cousino et al., 1998; Egan et al., 1999). Moreover, the C-terminal targeting information in *S. cerevisiae* TOM5 is not conserved in other TOM-TA proteins, since the TOM5 and TOM6 TMDs are not functionally interchangeable (Horie et al., 2003). The involvement of sequences upstream of the TA region is not unique to the targeting of TOM-TA proteins, since both the TA protein subunits of the translocon at the chloroplast outer membrane (i.e., TOC33 and TOC34) appear to rely on almost the entire protein for proper targeting (Chen and Schnell, 1997; Horie et al., 2003). One possible explanation for this is that the TA-protein subunits of the translocon of mitochondria and chloroplasts have more complex requirements in terms of their biogenesis: a multi-step process that begins with their targeting to the surface of the proper organelle, followed by their insertion into the lipid bilayer, and eventually their assembly into a functional multi-protein complex. Furthermore, all of these steps are considered to be dependent primarily on the proteins themselves, given their roles as receptor proteins. As such, it is reasonable that sequences in addition to those in their TA regions have a role(s) in the targeting of TOM-TA proteins, as well as performing other distinct functions such as insertion, assembly, stabilization, and turnover (Habib et al., 2003).

### Mutational analysis of the dibasic motif

Given that the targeting information in the TOM proteins is likely to be more complex, and not necessarily conserved between members of the TOM family (Figure 4B), we chose to focus on gaining a better understanding of the nature of the dibasic targeting motif in the second group of proteins. Toward this end, we carried out a mutational analysis of the dibasic motif and other adjacent sequences in the CTS. We focused initially on using the TraB protein as a template, since this protein has not been extensively characterized to date.

**Figure 5:**
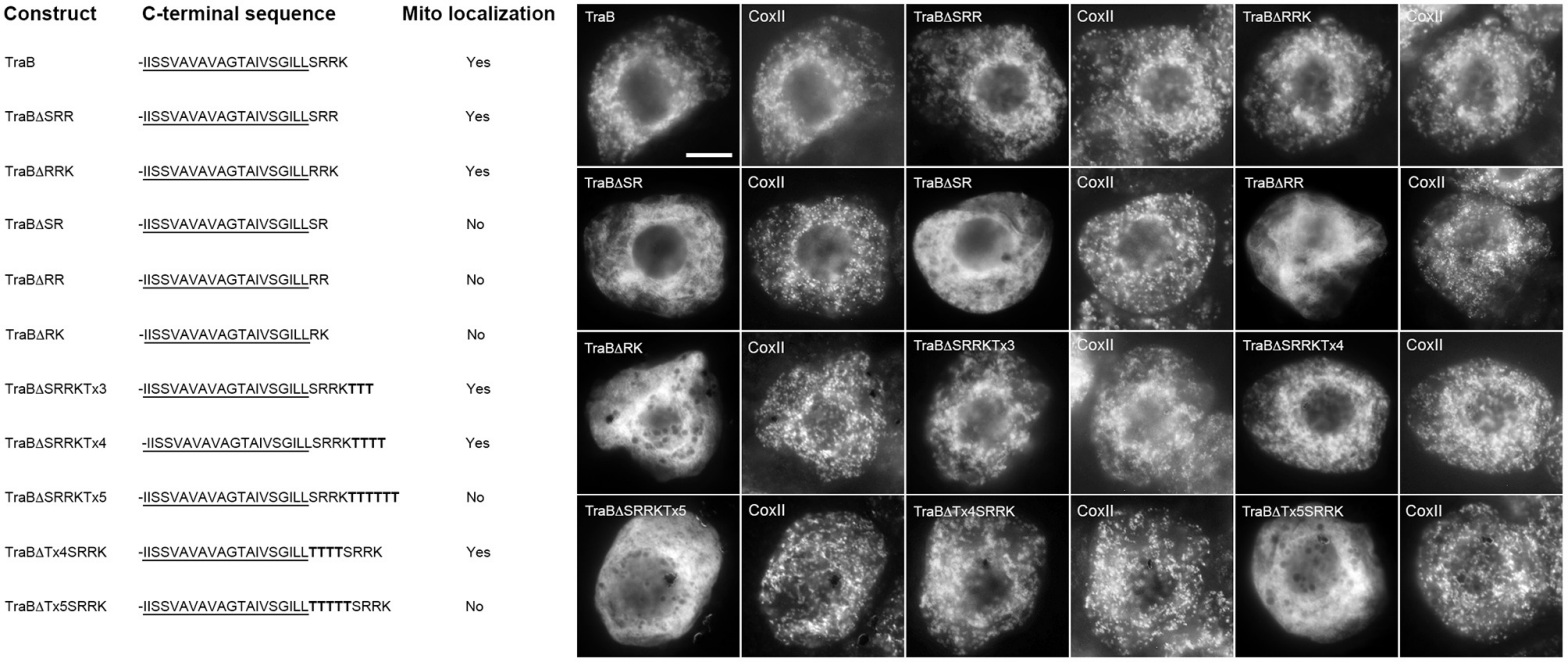
**Localization of various CTS mutant versions of the mitochondrial-TA protein TraB in BY-2 cells**. Shown on the **left** are schematic illustrations of wild-type and various CTS mutant versions of TraB and their corresponding localization (or lack thereof) to mitochondria in transformed BY-2 cells. The names of the mutant constructs represent the specific amino acids in their modified CTSs. All constructs possess an N-terminal-appended Myc-epitope tag. Shown for each construct is the corresponding C-terminal amino acid sequence, including putative TMD (underlined) and modified (or wild-type) CTS; additional amino acid residues inserted into the TraB CTS (i.e., threonines) are bolded. Mitochondrial localization (indicated as “Yes” or “No”) was assessed based on colocalization (or lack thereof) of the expressed protein and endogenous mitochondrial CoxII. Shown on the **right** in both are representative immuno-epifluorescence micrographs illustrating the localization of the various constructs shown on the **left**. Each micrograph is labeled with the name of the expressed Myc-tagged wild-type TraB or CTS mutant version of TraB, or endogenous CoxII. Bar = 10 μm.

The TraB CTS is -SRRK (Table 1), and we first examined the importance of the length and positioning of the charged residues within the CTS relative to the TMD and C terminus. As shown in Figure 5, while singular deletions of the C-terminal lysine (TraBΔSRR) or the serine at the -4 position (TraBΔRRK) had no apparent effect on the mitochondrial localization, deletion of the last two amino acids in the CTS, leaving just -SR-, resulted in the modified protein (TraBΔSR) being mislocalized. Similarly, amino acid deletions leaving just -RR- (TraBΔ RR) or -RK- (TraBΔRK) also resulted in mislocalization (Figure 5). These results indicate that a dibasic sequence alone is not sufficient for mitochondrial targeting of TraB, which is consistent with the previously published results for tung Cb5D (Hwang et al., 2004), whereby the dibasic sequence must be positioned within a CTS at least three amino acids in length.

To determine how far the dibasic motif could be positioned relative to the TMD or C terminus of TraB, threonine residues were added either before or after the CTS. As shown in Figure 5, mitochondrial targeting of TraB was preserved when three or four threonines were added to the end of its CTS (TraBΔSRRKTx3 and TraBΔSRRKTx4), but the addition of five threonines (TraBΔSRRKTx5) resulted in mislocalization. Similarly, the insertion of three threonines between the predicted border of the TMD and the CTS of TraB (TraBΔTx4SRKK) maintained mitochondrial targeting, but not when five threonines were inserted at this position (TraBΔTx5SRKK) (Figure 5). Together, these results and those presented previously on the impact of added threonine residues before or after the CTS in tung Cb5D (Hwang et al., 2004) reveal that the dibasic motif can tolerate being positioned at least four amino acids from either the TMD or C terminus. Our results also reveal that while a certain degree of sequence divergence exists in the CTSs of the mitochondrial-TA proteins that possess a dibasic motif, most of these proteins contain a serine residue between the predicted end of the TMD and the dibasic sequence (see Table 1), suggesting that this residue might be important for conveying the proper functional context for the dibasic targeting signal. Indeed, several other discrete targeting signal motifs appear to rely on adjacent so-called “accessory” or “secondary” amino acid residues for efficient function, including nuclear localization signals (Dussert et al., 2013), ER membrane retrieval motifs (Gidda et al., 2009), and peroxisomal matrix targeting signals (Kunze et al., 2011; Lingner et al., 2011).

**Figure 6:**
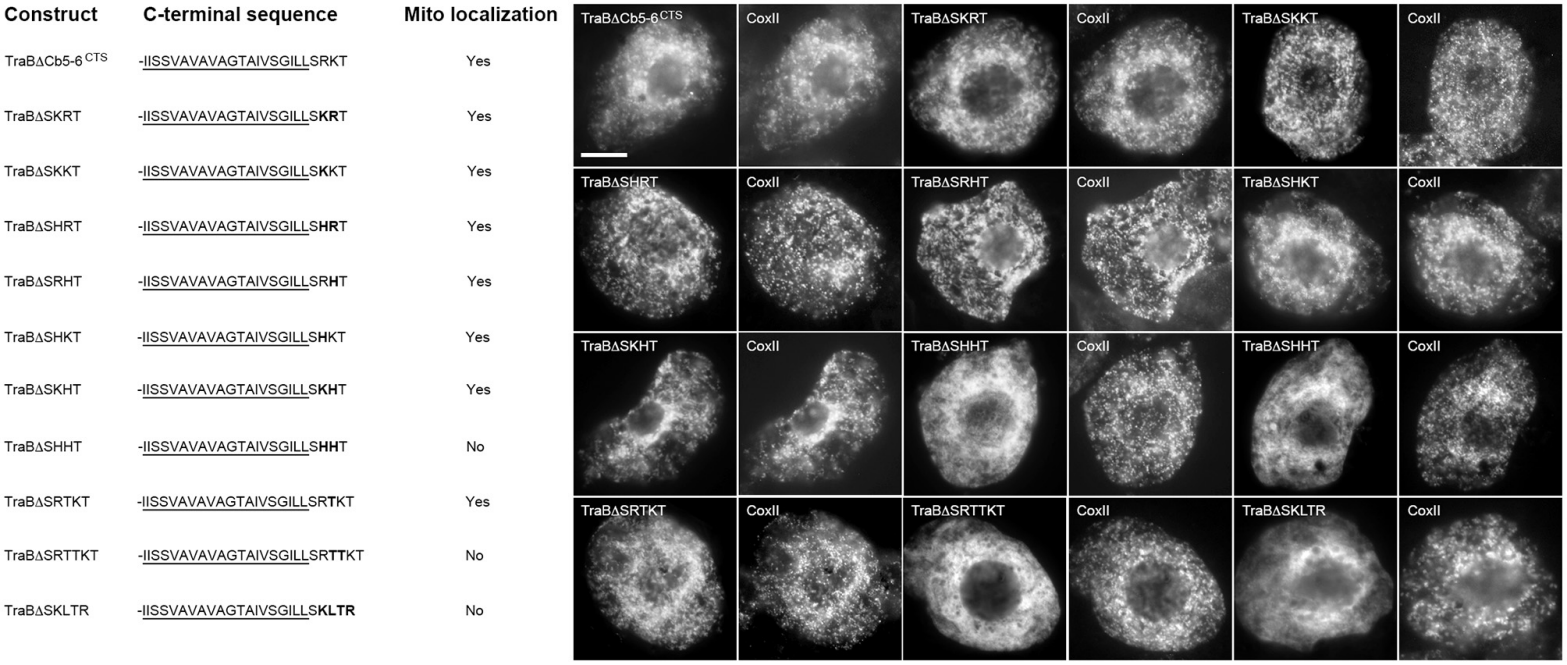
**Localization of various CTS mutant versions of the mitochondrial-TA hybrid protein TraBΔCb5-6^CTS^ in BY-2 cells**. Shown on the **left** are schematic illustrations of wild-type and various CTS mutant versions of the hybrid protein TraBΔCb5-6^CTS^ and their corresponding localization (or lack thereof) to mitochondria in transformed BY-2 cells. The names of the mutant constructs represent the specific amino acids in their CTSs. All constructs possess an N-terminal-appended Myc-epitope tag. Shown for each construct is the corresponding C-terminal amino acid sequence, including putative TraB TMD (underlined) and modified (or wild-type) Cb5-6 CTS; modified or additional amino acid residues inserted into the Cb5-6 CTS are bolded. Mitochondrial localization (indicated as “Yes” or “No”) was assessed based on colocalization (or lack thereof) of the expressed protein and endogenous mitochondrial CoxII. Shown on the **right** in both are representative immuno-epifluorescence micrographs illustrating the localization of the various constructs shown on the **left**. Each micrograph is labeled with the name of the expressed Myc-tagged TraBΔCb5-6^CTS^ or CTS mutant version of TraBΔCb5-6^CTS^, or endogenous CoxII. Bar = 10 μm.

We next investigated the identity of the basic residues in the dibasic sequence itself, and our template for these experiments was the chimera TraBΔCb5-6^CTS^, which as described above (Figure 4), consists of the TraB protein with its CTS replaced with the CTS of Cb5-6 (-SRKT). We chose this sequence since the CTS has just two basic amino acids. As shown in Figure 6, this -RK- dibasic sequence could be replaced by a variety of other combinations of basic amino acids without disrupting mitochondrial targeting. That is, arginine, lysine, and histidine were all acceptable at either the first or second position of the dibasic sequence, as long as it was paired with either another arginine or lysine residue. Only a dibasic sequence consisting of two histidine residues (i.e., TraBΔSHHT) abolished mitochondrial targeting (Figure 6).

We tested also whether the dibasic sequence in TraBΔCb5-6^CTS^ could tolerate an amino acid insertion between the two basic residues, similar to the CTS of PMD1 (-SKLR). As shown in Figure 6, insertion of a single threonine residue between the dibasic resides of TraBΔCb5-6^CTS^ (TraBΔSRTKT) had no effect on localization to mitochondria, whereas the insertion of two threonine residues (TraBΔSRTTKT) disrupted mitochondrial targeting. In line with these latter results, insertion of a threonine residue alongside the intervening leucine residue in the CTS of PMD1 (TraBΔSKLTR) also disrupted mitochondrial targeting (Figure 6). These data reinforce the notion that the dibasic motif does not have to be contiguous, and that the motif can tolerate one, but not two, amino acid residues in between the two basic residues.

**Figure 7:**
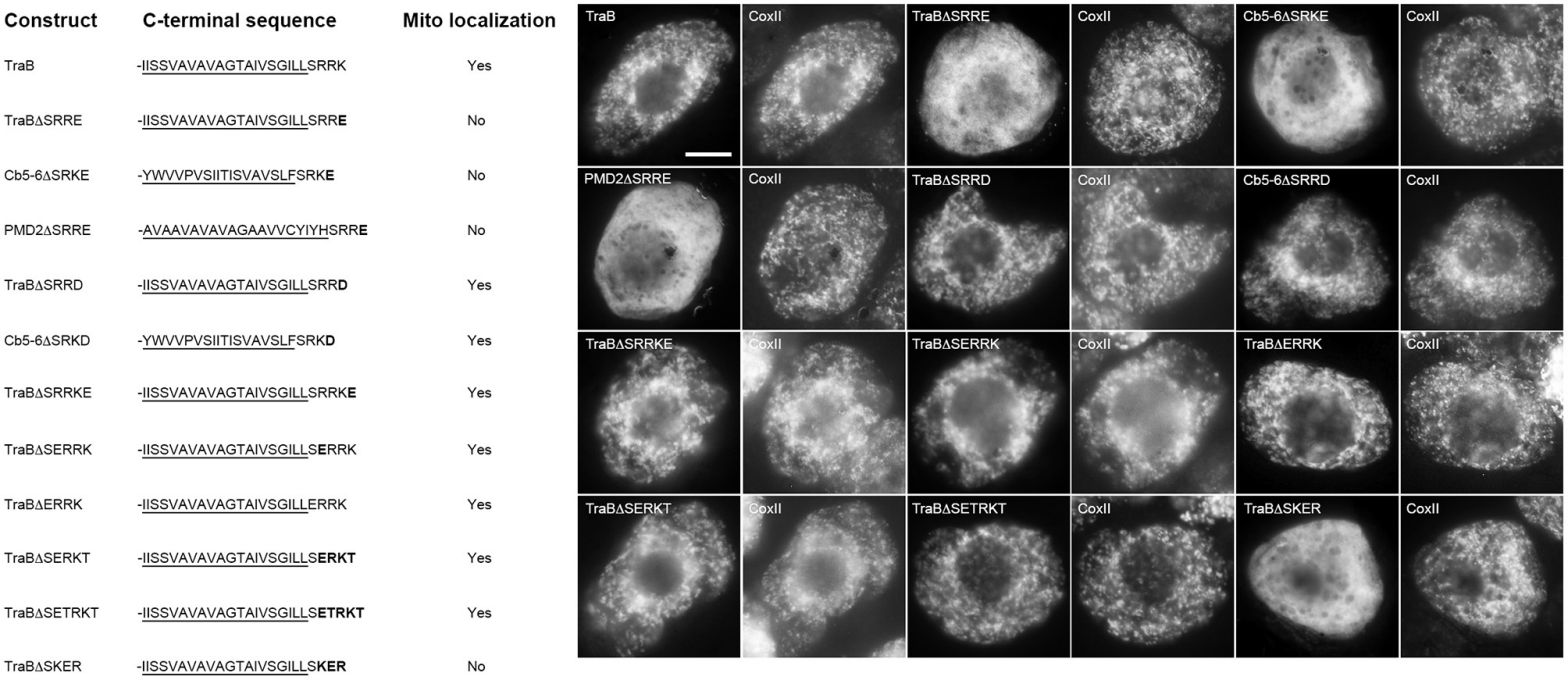
**Localization of various CTS mutant versions of mitochondrial-TA proteins in BY-2 cells**. Shown on the **left** are schematic illustrations of wild-type and/or various CTS mutant versions of TraB, Cb5-6, or PMD2 and their corresponding localization (or lack thereof) to mitochondria in transformed BY-2 cells. The names of the mutants represent the specific amino acids in their modified CTSs. All constructs also possess an N-terminal-appended Myc-epitope tag. Shown for each construct is the corresponding C-terminal amino acid sequence, including putative TMD (underlined) and modified (or wild-type) CTS from TraB, Cb5-6 and PMD2; modified amino acid residues in the protein's CTS are bolded. Mitochondrial localization (indicated as “Yes” or “No”) was assessed based on colocalization (or lack thereof) of the expressed protein and endogenous mitochondrial CoxII. Shown on the **right** in both are representative immuno-epifluorescence micrographs illustrating the localization of the various constructs shown on the **left**. Each micrograph is labeled with the name of the expressed Myc-tagged wild-type and/or CTS mutant version of TraB, Cb5-6, or PMD2 or endogenous CoxII. Bar = 10 μm.

Given the apparent importance of basic amino acid residues in the dibasic motif, and that, with the exception of APX-5, all of the mitochondrial-TA proteins that possess a dibasic motif also lack an acidic amino acid in their CTS (see Table 1), we examined next the influence of adjacent acidic residues on the dibasic motif. As shown in Figure 7, replacement of the C-terminal amino acid residue in the CTS of TraB, Cb5-6, and PMD2, with a glutamic acid (TraBΔSRRE, Cb5-6ΔSRKE, and PMD2ΔSRRE), which places this acidic residue immediately downstream of the dibasic sequence, abolished mitochondrial targeting. These results are consistent with those published previously for tung Cb5D, whereby “X” in the dibasic motif could not be a glutamate (Hwang et al., 2004). Replacement of the C-terminal amino acid residue in TraB or Cb5-6 with an aspartate residue (TraBΔSRRD and Cb5-6ΔSRKD), however, did not disrupt mitochondrial targeting (Figure 7). Moreover, the addition of a glutamic acid residue to the C-terminal end of the TraB CTS (TraBΔSRRKE), did not abolish targeting, nor did placement of a glutamic acid before the dibasic sequence in TraB (TraBΔSERRK or TraBΔERRK) (Figure 7). The results of the latter two constructs were complicated, however, by the fact that there are three basic residues in the CTS of TraB (-SRRK), and so it is not entirely clear which (or both) of the two pairs of basic residues serve as the targeting signal. To address this, we employed TraBΔCb5-6^CTS^ as an alternate template, since the CTS of this chimera consists of only a single dibasic sequence (i.e., -SRKT). As shown in Figure 7, insertion of a glutamic acid residue just before the dibasic sequence in the CTS of TraBΔCb5-6^CTS^ (TrabBΔSERKT) did not disrupt targeting to mitochondria. On the other hand, insertion of a glutamic acid residue between the dibasic amino acids in TraBΔCb5-6^CTS^ (TrabBΔSREKT) did abolish mitochondrial targeting (Figure 7).

Taken together, these data significantly extend our understanding of the dibasic motif by revealing that an acidic amino acid is tolerated when located upstream of the dibasic motif, but not when placed downstream or within the motif, although in the latter instance aspartic acid is tolerated at the downstream position.

### Bioinformatics analysis using the expanded dibasic motif identifies new OMM-TA proteins

Based on the mutational analysis described above (Figures 5–7), we developed an expanded version of the dibasic targeting signal motif that accounts for the sequence variability now known to be acceptable for TA protein sorting to mitochondria in plant cells: -R/K/H-X^{0,1≠E}^-R/K/H^{≠−H−H−or−H−X−H^−}-X^{0,1≠E}^X^{0,3}{CTS=3,8}^, whereby the dibasic sequence can be any two basic amino acid residues, other than two histidines, and can be contiguous or separated by any one amino acid residue, other than a glutamic acid. The dibasic sequence can be also positioned 0 to 4 amino acid residues away from the C terminus, as long as the residue immediately downstream of the dibasic sequence is not a glutamic acid. Finally, the motif is considered to function only in the context of a CTS that is 3–8 amino acid residues in length, although this criteria is not as strict since there are no fully reliable methods for predicting the end(s) of a TMD and thus the precise length of the CTS.

Using this new dibasic motif and also taking into consideration that OMM-TA proteins are traditionally defined as possessing a single C-terminal TMD of relatively moderate hydrophobicity and devoid of a cleavable N-terminal presequence (Borgese et al., 2003; Abell and Mullen, 2011), we performed a bioinformatics search of all the TA proteins predicted for the *A. thaliana* deduced proteome (Kriechbaumer et al., 2009; Pedrazzini, 2009; Dhanoa et al., 2010) and by doing so we identified a total of 32 proteins (Table 2). Among these proteins were all of the dibasic motif-containing OMM-TA proteins from Table 1. Interestingly, the list also included a protein annotated as a third isoform of MIRO (MIRO3) (Yamaoka and Leaver, 2008), as well as several other proteins that are annotated by SUBA or AmiGO to be localized to and/or function at mitochondria, including a rhomboid-like protein (At1g18600), a putative lipoprotein (At4g31030), and several unknown proteins (i.e., At1g72020, At4g38490, and At5g35470). In addition, a number of proteins (i.e., 18 of 32) in Table 2 are annotated by SUBA as being uncharacterized in terms of their intracellular localization.

**Table 2:**
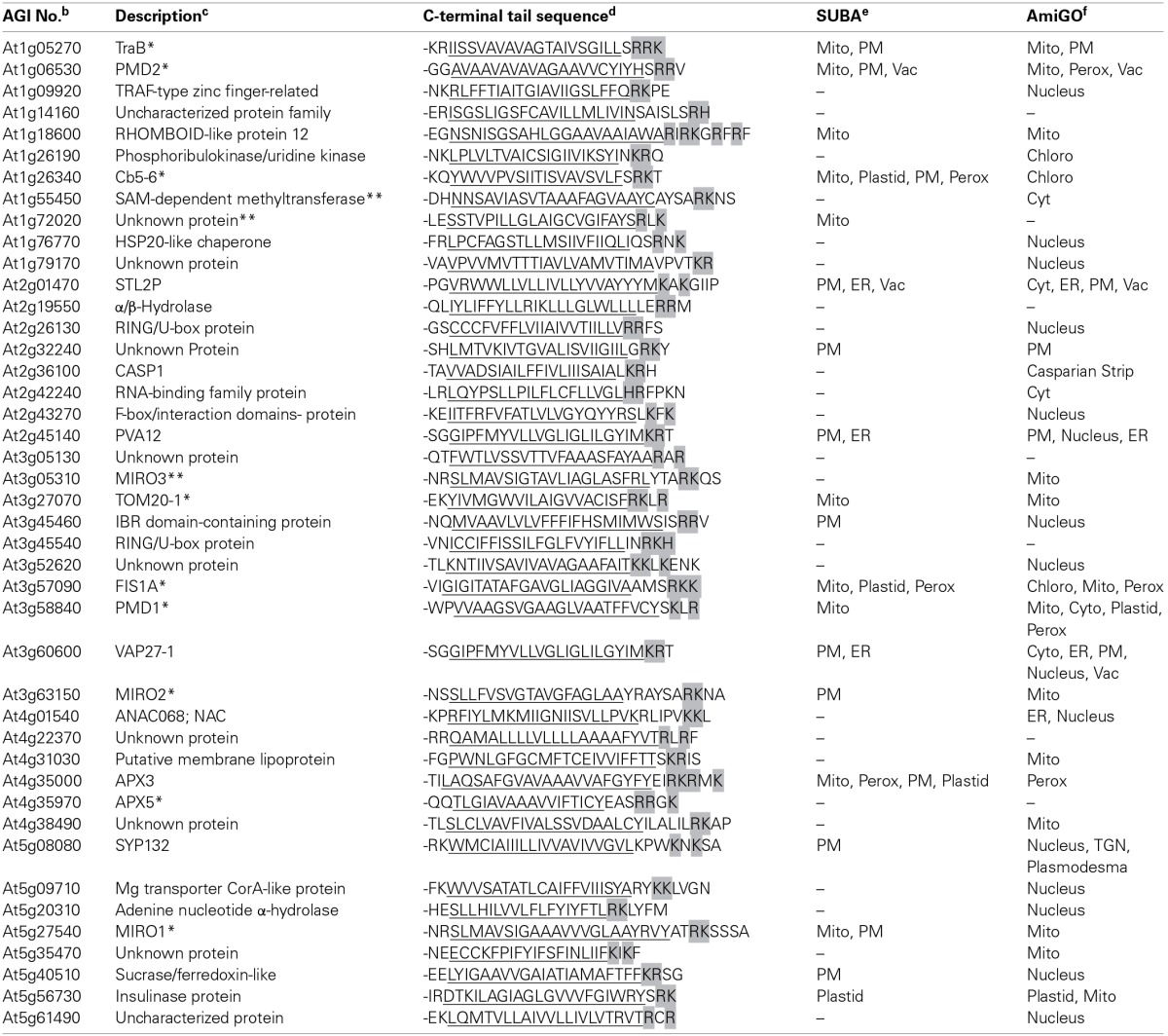
**Candidate *Arabidopsis* TA proteins containing a putative OMM dibasic targeting signal motif^a^**.

a All of the proteins listed are known or predicted to possess a TA orientation (Kriechbaumer et al., 2009; Pedrazzini, 2009; Dhanoa et al., 2010) and also contain a C-terminal mitochondrial dibasic targeting signal motif according to the results of the mutational analysis of selected TA proteins presented this study (see Figures 5–7). See text, including Materials and Methods, for additional details.

^b^AGI number represents the systematic designation given to each locus, gene, and its corresponding protein product(s) in TAIR. Proteins are listed in ascending order based on their AGI number.

^c^Common nomenclature of Arabidopsis TA proteins based on published literature and TAIR. Proteins indicated with an asterisk are also listed in Table 1; proteins indicated with two asterisks were experimentally characterized in terms of their intracellular localization and, for At1g55450, also TA orientation, membrane integration, and C-terminal-tail-dependent targeting; see **Figure 8** and text for additional details.

^d^Shown for each protein is its deduced C-terminal tail sequence, including its putative TMD (underlined), based on TOPCONS and visual inspection, and its downstream CTS. Shaded are the dibasic amino acid residues within the mitochondrial dibasic targeting signal motif (i.e., -R/K/H-X^{0,1≠E}^-R/K/H^{≠-H-H- or -H-X-H-}^-X^{0,1≠E}^X^{0,3}{CTS=3,8}^) found in the CTS of all the proteins shown.

^e^Shown for each protein is its intracellular localization(s) based on published proteomics and/or GFP localization results presented at SUBA3 (The SUBcellular localization database for Arabidopsis proteins) (Tanz et al., 2013). Abbreviations: ER, endoplasmic reticulum; Mito, mitochondria; Perox, peroxisome; PM, plasma membrane; Vac, vacuole.

^f^Shown for each protein is “cellular component” ontology, i.e., where a gene product is located in is a subcompartment of a particular cellular component, based on the AmiGO search tool at the Gene Ontology database (http://www.geneontology.org) (Ashburner et al., 2000). Abbreviations: Cyt, cytosol; TGN, trans-Golgi network; see also above (footnote ^e^).

**Figure 8:**
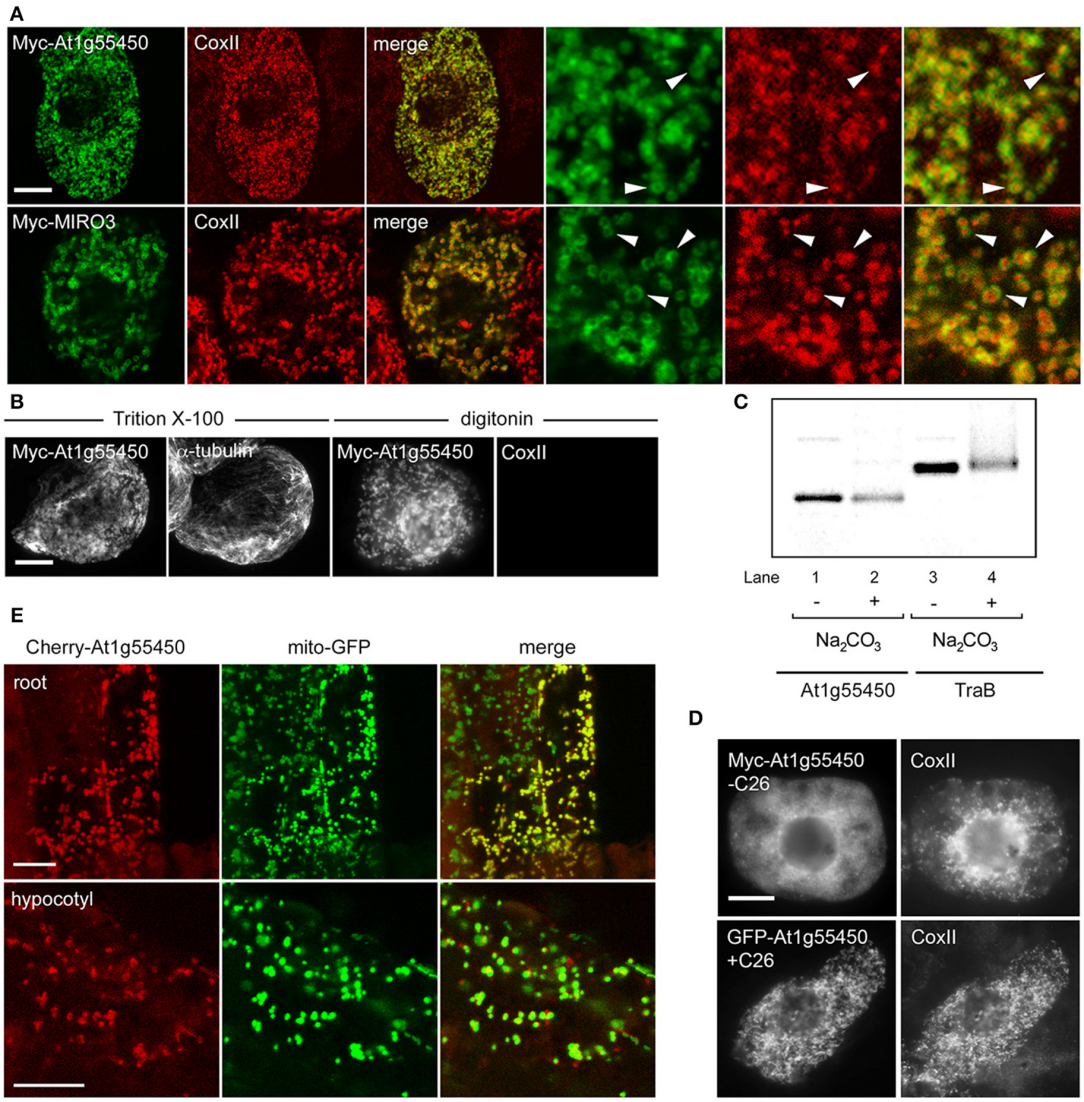
**Localization, topology, membrane insertion, and C-terminal targeting-signal analysis of a novel OMM-TA protein, At1g55450. (A)** Representative CLSM micrographs illustrating the localization of N-terminal Myc-tagged At1g55450 or MIRO3 to the OMM in BY-2 cells. Cells were processed for immunofluoescence CLSM as in Figure 1. Shown in the three panels on the right are images corresponding to a portion of the cell at higher magnification. Solid arrowheads indicate examples of the torus-shaped fluorescent structures containing the transiently-expressed protein delineating the spherical structures attributable to endogenous CoxII. **(B)** Topological mapping of Myc-At1g55450 in differential-permeabilized BY-2 cells. Cells transiently-transformed with N-terminal Myc-tagged At1g55450 were formaldehyde fixed and permeabilized with either Triton X-100 or digitonin, and then cells were processed for immuno-epifluorescence microscopy, as described in Figure 2. **(C)** Insertion of At1g55450 into mitochondrial membranes *in vitro*. Isolated pea mitochondria were incubated with *in vitro* synthesized Myc-At1g55450 (lanes 1 and 2) or, for comparative purposes, Myc-TraB (lanes 3 and 4), and then resuspended (+) or not (−) in alkaline Na_2_CO_3_. Equivalent amounts of each alkaline Na_2_CO_3_- or mock-extracted sample were then subjected to SDS-PAGE and phosphoimaging. **(D)** Representative immuno-epifluorescence micrographs illustrating the localization of a C-terminal mutant or GFP fusion of At1g55450 in BY-2 cells. Each micrograph is labeled with the name of either the transiently-expressed Myc-tagged C-terminal mutant or GFP fusion protein or endogenous CoxII. The name of each construct includes the number of amino acid residues that were either deleted from the C terminus of Myc-tagged At1g55450 (−C26) or fused to the C terminus of GFP (+C26). **(E)** Representative CLSM micrographs illustrating the localization of the Cherry-At1g55450 fusion protein to mitochondria in living transgenic *A. thaliana* seedlings co-expressing the mitochondrial marker protein mito-GFP. Labels above the panels indicate the name of the co-expressed protein and labels in panels on the left indicate the seedling tissue type. Note in the top row that not all root cells expressed the Cherry-At1g55450 fusion protein. Bars in **(A** and **B)** and **(D** and **E)** = 10 μm.

To determine whether any of these proteins represented *bona fide* OMM-TA proteins, we focused on At1g55450, which is of unknown function, but, based on information provided at TAIR, does contain an S-adenosyl-L-methionine (SAM)-dependent methyltransferase domain in its N-terminal region. To investigate whether At1g55450 was localized to mitochondria, we transiently expressed an N-terminal Myc-tagged version in BY-2 cells and visualized the cells using immunostaining and CLSM. This analysis revealed that the protein localized to ring-shaped structures that encircled the punctate immunofluorescence pattern of endogenous CoxII (Figure 8A), similar to the localization pattern of other OMM-TA proteins presented in Figure 1. Notably, similar OMM localization was also observed for another protein from Table 2, namely MIRO3 (Figure 8A).

We next determined whether the unknown protein At1g55450 adopted a TA protein topology, with the N terminus orientated toward the cytoplasm. As shown in Figure 8B, the Myc epitope appended to the N-terminus of the At1g55450 protein (Myc-At1g55450) was indeed detectable in either Triton X-100- or digitonin-pemeabilized BY-2 cells (Figure 8B), similar to the other TA proteins presented in Figure 2. To confirm that this protein could integrate into mitochondrial membranes, we performed *in vitro* mitochondrial targeting studies. The protein was first synthesized and radiolabeled using *in vitro* transcription/translation reactions then incubated with isolated mitochondria. Following incubation, mitochondria were washed with alkaline sodium carbonate to remove peripherally-associated proteins, then membranes were pelleted by centrifugation. As shown in Figure 8C, Myc-At1g55450, as well as Myc-TraB, was resistant to alkaline sodium carbonate extraction, which operationally defines both of these proteins as integral membrane proteins.

Consistent also with the other dibasic-motif-containing mitochondrial-TA proteins, deletion of the C-terminal TA region of Myc-At1g55450 (Myc-At1g55450-C26) abolished mitochondrial targeting, while fusion of this same region to GFP (GFP-At1g55450+C26) resulted in localization to mitochondria (Figure 8D). As such, the TA region was both necessary and sufficient for targeting to mitochondria, similar to the other dibasic-containing proteins (Figure 3). Finally, stable expression of Cherry-tagged At1g55450 in transgenic *A. thaliana*, also transformed with a mitochondrial marker fusion protein consisting of the *Nicotiana plumbaginifolia* β-ATPase N-terminal mitochondrial presequence fused to GFP [(Mito-GFP) (Logan and Leaver, 2000)], resulted in colocalization of the two proteins in living cells in roots and hypocotyls (Figure 8E). Taken together, these data clearly define At1g55450 as a new OMM-TA protein in plants. Whether the other proteins in Table 2 are also *bona fide* members of the OMM subproteome remains to be determined.

## Conclusions

While in recent years considerable progress has been made toward understanding the biogenesis of TA proteins in yeast and mammals, relatively little is known about how these proteins are properly partitioned within plant cells. Compounding this problem is the added complexity associated with how TA proteins must differentiate between not only mitochondria and ER (as they do in yeast and mammals), but also, unique to plant cells, plastids (reviewed in Abell and Mullen, 2011; Kim and Hwang, 2013; Lee et al., 2014). One main reason for this paucity of knowledge is that so few plant TA proteins have been identified, let alone characterized in terms of their organelle-specific targeting information and/or the cellular machinery involved in their membrane import and assembly. Moreover, at least some of the plant TA proteins examined to date appear to localize to different compartments depending on cell type and/or experimental condition (Scott et al., 2006; Maggio et al., 2007; Lingard et al., 2008; Zhang and Hu, 2009; Aung and Hu, 2011; Sun et al., 2012; Ruberti et al., 2014), implying that the targeting of TA proteins in plants is even more complex, and substantially different from the targeting of TA proteins in yeast and mammals.

Herein we describe an important step toward a better understanding of plant TA protein biogenesis by showing that a dibasic targeting signal motif that was previously identified in a mitochondrial isoform of Cb5 (Hwang et al., 2004) is also present in a number of other mitochondrial-TA proteins in plants, and, this motif is absent in all but one of the TOM-TA proteins. We also showed that the motif is far more divergent than previously defined, including the acceptable combination of basic amino acids, the contiguous nature of the dibasic sequence, the length of the CTS, and the relative position of the dibasic sequence within the CTS (Figures 5–7). Furthermore, we utilized this motif to identify a number of new, putative OMM-TA proteins (Table 2), including a protein that while annotated to be of unknown function, is predicted to possess an N-terminal SAM-dependent methyltransfase domain and thus might operate in corresponding manner at the cytosolic surface of the mitochondrion. Of course, one important caveat of this study is that the consensus targeting sequence we define here for OMM-TA proteins, like most other discrete sequence-specific targeting signal motifs (e.g., PTS1, -HDEL, and dilysine ER retrieval signals, etc.), is probably highly context dependent. As such, it is not unreasonable to expect that additional variations of the motif exist and that these operate in a cooperative manner with targeting elements conveyed by the physicochemical and perhaps sequence-specific properties within the upstream regions, particularly within the TMD, of different proteins. Hence, why we observed at least some contradictions between the functional definition of the dibasic targeting motif in the proteins examined in this study and that reported previously for Cb5 (Hwang et al., 2004), and why some of the proteins listed in Table 2 may target not only to mitochondria, but also to other organelles (i.e., peroxisomes or chloroplasts), or perhaps do not target to mitochondria. Regardless, the future study of these proteins, including those that perhaps localize to other organelles, will still serve to provide a better understanding of TA protein targeting pathways in plants in general.

Another critical aspect of plant mitochondrial-TA protein biogenesis that remains obscure is the biogenesis of TA-TOM proteins, which, based on the results of this study (Figures 3, 4), appear to involve targeting information that is distinct from that of dibasic-motif-containing mitochondrial-TA proteins. Like other TA proteins that are components of the protein translocons at the ER or chloroplasts, TA-TOM proteins have been proposed to have evolved during a process whereby TA proteins, due to their relatively simple structure, inserted into membranes in early cells with minimal assistance and then over time mediated the subsequent insertion of more complex proteins (Borgese and Righi, 2010). As such, one possibility is that, similar to the scenario proposed recently for mitochondrial-TA proteins in yeast (Krumpe et al., 2012) and TA-TOC proteins (Dhanoa et al., 2010), the inherent unique lipid composition of the OMM, possibly in conjunction with cytoplasmic chaperones [or perhaps without them (Kriechbaumer et al., 2009)], might specify the targeting and insertion of TA-TOM proteins in plant cells. Alternatively, or in addition to, the targeting and insertion specificity of plant TOM-TA proteins, and perhaps mitochondrial-TA proteins in plants in general, might involve a modified version of the GET pathway, which, as described in the Introduction, normally mediates ER-TA protein biogenesis (Denic, 2012). For instance, *A. thaliana* possess three putative orthologs of the yeast GET3 protein (Abell and Mullen, 2011; Duncan et al., 2013), including one that, like its yeast and mammalian counterparts, localizes to the cytoplasm and is presumed to play a role in directing TA proteins to the ER (Abell and Mullen, 2011; Duncan et al., 2013). The other two *A. thaliana* GET3 proteins, however, localize to the chloroplast stroma (Rutschow et al., 2008; Ferro et al., 2010) or OMM (Duncan et al., 2013), where they are thought to play an analogous role in TA protein biogenesis in the respective organelle (Duncan et al., 2013). However, the observation that plants appear to lack homologs to the other protein components of the GET machinery (i.e., GET1, 2, 4, and 5), has led to the suggestion that GET3 proteins in plants, including mitochondrial GET3, mediate TA protein biogenesis in a unique manner compared to the GET pathway in yeasts and mammals (Duncan et al., 2013). Future studies are required to test this hypothesis, as well as determine the potential role of membrane lipids and/or other sorting machinery, such as the TOM complex or otherwise [e.g., SAM (Stojanovski et al., 2007)], in the biogenesis of mitochondrial-TA proteins in plant cells.

## Author contributions

Naomi J. Marty, Yeen Ting Hwang, Howard J. Teresinski, and Eric A. Clendening generated plasmid DNA constructs and/or performed subcellular localization studies in BY-2 cells; Satinder K. Gidda also assisted with subcellular localization studies and generated the *A. thaliana* plants stably expressing Cherry-At1g55450 and mito-GFP; Elwira Sliwinska performed the microscopic analysis of transgenic *A. thaliana* seedlings; Daiyuan Zhang generated selected plasmid DNA constructs; Ján A. Miernyk performed the isolated mitochondria *in vitro* membrane insertion experiments; Glauber C. Brito, and David W. Andrews provided TAMP and participated in the bioinformatics analysis of predicted *A. thaliana* OMM-TA proteins; and John M. Dyer and Robert T. Mullen designed the study and wrote the paper.

### Conflict of interest statement

The authors declare that the research was conducted in the absence of any commercial or financial relationships that could be construed as a potential conflict of interest.
